# The transcription factor ZFP64 promotes activity-dependent synapse elimination during postnatal cerebellar development

**DOI:** 10.1016/j.isci.2025.112746

**Published:** 2025-05-26

**Authors:** Jianling Zhang, Takaki Watanabe, Taisuke Miyazaki, Miwako Yamasaki, Kohtarou Konno, Yuto Okuno, Kyoko Matsuyama, Takayuki Noro, Masahiko Watanabe, Naofumi Uesaka, Masanobu Kano

**Affiliations:** 1 Department of Neurophysiology, Graduate School of Medicine, The University of Tokyo, Tokyo 113-0033, Japan; 2 International Research Center for Neurointelligence (WPI-IRCN), The University of Tokyo, Tokyo 113-0033, Japan; 3 Advanced Comprehensive Research Organization (ACRO), Teikyo University, Tokyo 173-0003, Japan; 4 Department of Anatomy, Graduate School of Medicine, Hokkaido University, Sapporo 060-8638, Japan; 5 Department of Cognitive Neurobiology, Graduate School of Medical and Dental Sciences, Institute of Science Tokyo, Tokyo 113-8510, Japan

**Keywords:** Molecular physiology, Neuroscience, Developmental biology

## Abstract

Eliminating redundant synapses formed around birth is essential for shaping functionally mature neural circuits during postnatal development. Each Purkinje cell (PC) in the neonatal mouse cerebellum receives synaptic inputs from multiple climbing fibers (CFs). Only one CF is strengthened and extends its innervation over PC dendrites, whereas the other CFs are eventually pruned during postnatal development. These events are believed to require proper gene expression, but the underlying mechanisms are not yet understood. Here, we report that the transcription factor ZFP64 in PCs mediates part of CF synapse elimination events presumably downstream of P/Q-type voltage-dependent Ca^2+^ channels (P/Q-VDCCs). PC-specific knockdown (KD) of ZFP64 during postnatal development delayed the elimination of redundant CF synapses and the dendritic extension of CF innervation. The KD of semaphorin 3A (Sema3A) in PCs partially restored the effects of ZFP64 or P/Q-VDCC KD. We propose that ZFP64 promotes developmental CF synapse elimination by regulating Sema3A expression.

## Highlights

• The transcription factor ZFP64 in PCs mediates a part of CF synapse elimination
• PC-specific KD of ZFP64 delays developmental elimination of redundant CF synapses
• ZFP64-KD in PCs delays dendritic extension of CFs with enhanced PF-PC transmission
• ZFP64 exerts these effects presumably downstream of P/Q-VDCC by regulating Sema3A

## Graphical abstract

**Figure:**
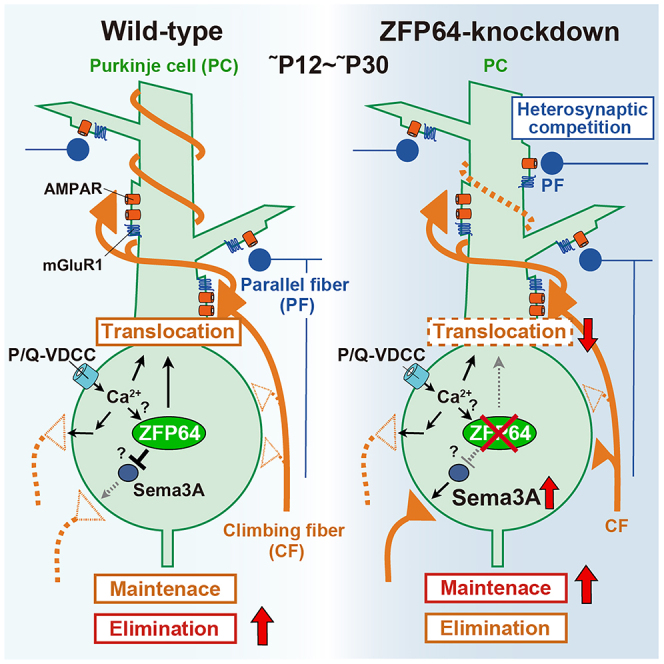

## Introduction

The nervous systems of neonatal animals contain exuberant, redundant synapses. During postnatal development, functionally mature neural circuits are established by strengthening essential synapses and weakening and eventually eliminating redundant ones,^1^^,^^2^^,^^3^ which is known as synapse elimination and is widely seen in various regions of the central and peripheral nervous systems. Among them, the postnatal development of climbing fiber (CF) to Purkinje cell (PC) synapses in the cerebellum has been a representative model of synapse elimination.^4^ In the neonatal mouse cerebellum at around postnatal day 3 (P3), each PC receives synaptic inputs on the soma from more than five CFs with similar strengths.^5^ Inputs from a single CF become progressively stronger on the soma, relative to the other CFs, in each PC by P7 (termed “functional differentiation”), establishing a single “winner” CF and the other “loser” CFs. Then, the winner CF extends its innervation territory along PC dendrites from around P9 (termed “CF translocation”).^6^ In parallel, synapses of the loser CFs are eliminated from the soma in two distinct processes, from around P7 to around P11 occurring independently of the formation of synapses from parallel fibers (PFs), the other excitatory inputs to PCs (termed “early phase of CF elimination”) and from around P12 to around P17 proceeding critically dependent on PF-PC synapse formation (termed “late phase of CF elimination”).^7^^,^^8^ While the mono-CF innervation pattern of each PC is established at around P17, the CF translocation continues, and excess PF synapses are eliminated from the mid portions of PC dendrites until around P30, when mature CF and PF synaptic wirings on PCs are established.^9^

Previous studies show that neural activity is crucial for CF synapse elimination. Two canonical pathways in PCs initiated by the activation of type 1 metabotropic glutamate receptor (mGluR1) and P/Q-type voltage-dependent Ca^2+^ channel (P/Q-VDCC/Ca_v_2.1 encoded by *Cacna1a*) have been identified to play pivotal roles. The mGluR1^10^ and its downstream molecules, including Gαq,^11^ phospholipase Cβ4 (PLCβ4)/PLCβ3,^12^^,^^13^ and protein kinase Cγ (PKCγ)^14^ are essential mainly for the late phase of CF elimination.^4^ This mGluR1-activated signaling cascade in PCs produces brain-derived neurotrophic factor (BDNF)^15^ and semaphorin 7A (Sema7A)^16^ which retrogradely act on CFs to eliminate loser CFs. The mGluR1 signaling cascade in PCs is suggested to be driven by neural activity along the mossy fiber (MF)-granule cell (GC)-PF-PC circuit,^5^^,^^17^^,^^18^ involving the AMPA-type glutamate receptor (AMPAR) at MF to GC synapses.^18^ In contrast, the P/Q-VDCC in PCs is mainly activated by CF synaptic inputs^19^ and is critical for the functional differentiation, the CF translocation, and the early phase of CF elimination.^19^^,^^20^ P/Q-VDCCs in PCs also regulate competitive excitatory synaptic wiring between CFs and PFs on PCs.^21^ The immediate-early gene *Arc/Arg3.1* (*Arc*), a molecule regulating AMPA receptor trafficking,^22^ has been shown to facilitate the late phase of CF elimination downstream of P/Q-VDCC.^23^ Moreover, GABAergic inhibition from basket cells to PCs regulates CF synapse elimination from around P10 to P12 by controlling Ca^2+^ transients in PCs through P/Q-VDCC.^24^^,^^25^^,^^26^ These results indicate that P/Q-VDCC and its downstream signaling in PCs are essential for almost all the processes for establishing CF and PF synaptic wiring on PCs.

Neural activity in PCs is thought to induce gene expression to cause persistent changes in neuronal connectivity, such as CF synapse elimination. We focused on transcription factors whose genes were identified to be expressed in PCs during postnatal development by our microarray analysis.^16^ We found that *Zfp64*, a gene encoding the transcription factor Zinc finger protein 64 (ZFP64) that belongs to the C2H2-type zinc finger family^27^^,^^28^ is involved in establishing CF to PC synaptic wiring. The results to be presented show that ZFP64 functions presumably downstream of P/Q-VDCC in PCs during postnatal development until around P20 and plays pivotal roles in the elimination of redundant CF synapses, dendritic translocation of the winner CF, and competitive expansion of PF innervation.

## Results

### Knockdown of *Zfp64* in PCs impairs CF synapse elimination

**Figure 1:**
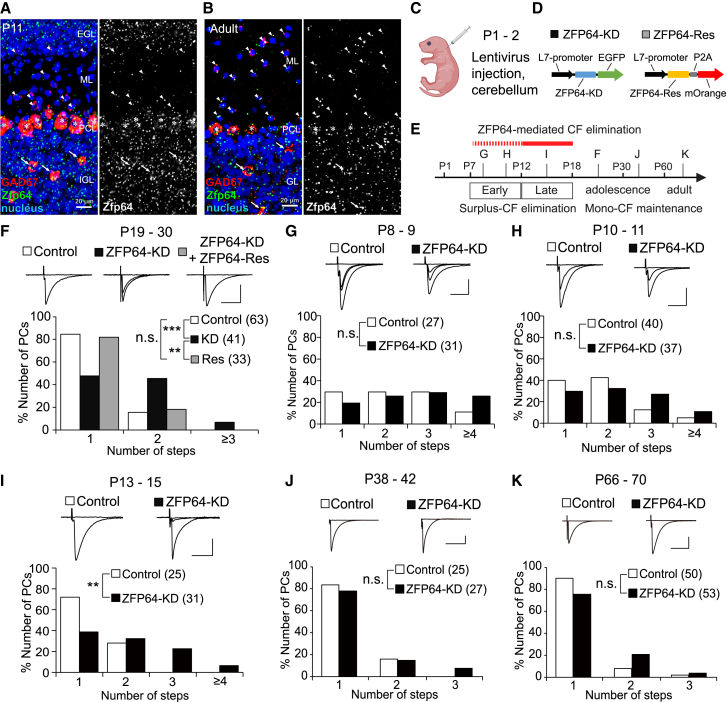
ZFP64-KD in PCs disrupts CF synapse elimination (A and B) Fluorescent *in situ* hybridization images of a cerebellar slice from a P11 (A) and a 3 to 4-month-old (B) wild-type mouse. ZFP64 mRNA (green) was broadly detected in GABAergic neurons expressing GAD67 mRNA (red), including PCs (asterisks), molecular layer interneurons (arrowheads), and Golgi cells (arrows), and in granule cells. Blue signals indicate nuclear stainings. ML, molecular layer; PCL, Purkinje cell layer; GL, granular layer; EGL, External granular layer; IGL, Internal granular layer. Scale bar, 20 μm. (C and D) Schema of lentivirus injection into the cerebellum at P1-2 and vector constructs for ZFP64-KD and ZFP64-Rescue (Res). (E) Time course of CF-PC synapse development showing the time points of data sampling for F to K. (F–K) CF innervation of PCs during P19-30 (F), P8-9 (G), P10-11 (H), P13-15 (I), P38-42 (J), and P66-70 (K). (Upper panels) Representative traces of CF-EPSCs recorded from GFP-negative untransfected control PCs (white, F–K), GFP-positive ZFP64-KD PCs (black, F–K), and a GFP/mOrange-positive ZFP64-KD/Res PC (gray, F). Holding potential, −10 mV. Scale bars, 1 nA and 20 ms. (Lower panels) Frequency distribution histograms for the number of CFs innervating individual PCs. The sample numbers of PCs are shown in parentheses. ∗∗∗*p* < 0.001, ∗∗*p* < 0.01, n.s. *p* > 0.05. Statistical tests and values are: (F) Kruskal-Wallis test, *H*_(2)_ = 25.0, *p* < 0.001, followed by Steel-Dwass test, *p* < 0.001 (control vs. ZFP64-KD), *p* = 0.873 (control vs. ZFP64-Res), and *p* = 0.0019 (ZFP64-KD vs. ZFP64-Res); (G–K) Mann-Whitney *U* test, *p* = 0.179 (G), *p* = 0.099 (H), *p* = 0.0035 (I), *p* = 0.513 (J), and *p* = 0.056 (K).

Among genes for transcription factors expressed in PCs during postnatal development, according to our PC-specific microarray data in L7-GFP transgenic mice,^16^ we focused on *Zfp64* encoding the transcription factor ZFP64 that has 3 isoforms (ZFP64 isoform 1, 2, and 3; registered in NCBI database) (Figure S1A). While *Zfp64* signals in PCs were relatively low when normalized, the expression of isoform 1 was consistently detected from P4 to P15, but the expression of isoforms 2 and 3 was hardly detectable (Table S1). We performed the fluorescent *in situ* hybridization analysis of *Zfp64* against isoform 1 in the cerebellum of P11 and adult (3-4-month-old) mice (Figures 1A and 1B). We found widespread expression of *Zfp64* in cells in the PC, granular, and molecular layers except for interneurons in the molecular layer at P11 (Figure 1A). In adulthood, *Zfp64* was expressed in almost all cell types in the cerebellum (Figure 1B). These data suggest that ZFP64 isoform 1 is a potential transcription factor for regulating gene expression in PCs during postnatal development.

We engineered two ZFP64-KD vectors, ZFP64-KD1 and -KD2, carrying the “miRNA-1” targeting on a shared part of the isoform 1 and 2 and the “miRNA-2” targeting on the isoform 1, respectively (Figure S1B). We tested the efficacy of the KD vectors in HEK293T cells and found that the KD1 and KD2 effectively suppressed the expression of ZFP64 isoform 1 but did not affect the expression of the rescue construct encoding the KD1/2-resistant ZFP64 isoform 1 (Figures S1B–S1D). In the following experiments, we used a mixture of KD1 and KD2 (hereafter termed “ZFP64-KD”) to ensure sufficient suppression of ZFP64 expression in PCs *in vivo* using two KD vectors targeting different portions of ZFP64-isoform 1.

First, we examined the effect of ZFP64-KD in PCs during early postnatal development on CF innervation in adolescence. We injected ZFP64-KD into the cerebellum of neonatal mice at P1-2 (Figures 1C and 1D). After the virus-injected mice grew to P19-30 (Figures 1E, 1F, and S2A), we prepared acute cerebellar slices from them, performed whole-cell recordings from EGFP-positive PCs with ZFP64-KD and EGFP-negative control PCs, and recorded excitatory postsynaptic currents (EPSCs) evoked by stimulating a CF. To stimulate CFs innervating the PC under recording, a stimulating electrode composed of a patch pipette filled with the external solution was placed in the granule cell layer underneath the recorded PC. To search all CFs innervating the recorded PC, we moved the stimulation pipette systematically around the PC soma and increased the stimulus intensity gradually at each stimulation site from 0 to 100 μA. We counted the number of discrete CF-EPSC steps and estimated the number of CFs innervating the PC under recording. We found that the percentage of PCs innervated by multiple CFs was significantly higher in ZFP64-KD PCs than in control PCs (Figure 1F). In contrast, when the RNAi-resistant form of ZFP64 isoform 1 (ZFP64-Res) was co-expressed in ZFP64-KD PCs, the percentage of multiply innervated PCs was the same as that of control PCs (Figure 1F), excluding the possibility of off-target effects of ZFP64-KD. Moreover, the expression of ZFP64-Res alone and the induction of possible overexpression of ZFP64 isoform 1 in PCs caused no significant effect on CF innervation at P11-14 when compared to control PCs (Figures S2B and S2C), suggesting that excess ZFP64 isoform 1 in PCs did not accelerate CF synapse elimination. These results indicate that the elimination of redundant CF synapses was impaired in ZFP64-KD PCs during postnatal development until P19.

### ZFP64 is mainly involved in the late phase of CF elimination

Elimination of surplus CFs from individual PCs occurs in two distinct developmental stages: the early phase (around P7 to around P11) and the late phase (around P12 to around P17) (Figure 1E).^5^ To determine when CF synapse elimination was impaired in ZFP64-KD PCs, we examined CF innervation at various stages of postnatal development until adulthood. We found no significant difference in the degree of multiple CF innervation between ZFP64-KD and control PCs at P8-9 and P10-11 (Figure 1G and 1H). In contrast, during P13 to P15 (Figure 1I) the degree of multiple CF innervation became significantly higher in ZFP64-KD PCs than in control PCs, suggesting that the late phase of CF elimination was impaired by ZFP64-KD in PCs. Similar impairment of CF synapse elimination at P12-14 was observed in PCs with ZFP64 isoform 1 KD by transfection of ZFP64-KD2 alone (Figures S1A and S2D), confirming that the isoform 1 in PCs is involved. This led to the persistent multiple CF innervation in ZFP64-KD PCs from P19 to P30. However, contrary to our expectations, no significant difference was found in the number of CF-EPSC steps between ZFP64-KD and control PCs from P38 to P42 (Figure 1J). The degree of multiple CF innervation was also at the same level between ZFP64-KD and control PCs from P66 to P70 (Figure 1K). These results indicate that the inhibitory effect of ZFP64-KD on CF synapse elimination was transient from around P12 to around P30, and the CF innervation pattern became normal in adolescence in ZFP64-KD PCs (Figure 1E). To check whether ZFP64-KD in PCs induces changes in CF to PC excitatory synaptic transmission, we analyzed the kinetics of CF-EPSCs for CFs in mono-innervated PCs (CF-mono), and the strongest CFs (CF-multi-S) and weaker CFs (CF-multi-W) in multiply innervated PCs (Table S2). We found no significant differences in the amplitude, rise time, and decay time constant for the three types of CFs between ZFP64-KD and control PCs during P8-11, P13-15, and P19-30 (Table S2). Moreover, the sum of multiple CF-EPSC amplitudes in individual PCs (total CF-EPSC amplitude), the two parameters representing the disparity among CF-EPSC amplitudes in individual PCs (Disparity ratio and Disparity index), and the paired-pulse ratio exhibited no significant differences between ZFP64-KD and control PCs (Table S3). These results suggest that ZFP64 is mainly involved in the late phase of CF elimination but does not contribute, if any, to the strengthening of a single CF and the basic properties of CF to PC excitatory transmission.

### ZFP64 acts presumably downstream of the P/Q-VDCC-mediated signaling pathway for CF synapse elimination

**Figure 2:**
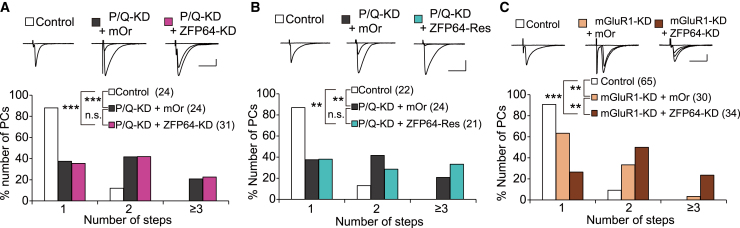
ZFP64 acts presumably downstream of P/Q-VDCC for CF synapse elimination (A–C) Representative CF-EPSC traces (upper) and frequency distribution histograms showing the number of CFs innervating each PC (lower) during P19-30 for untransfected control PCs (white), P/Q-KD + mOrange PCs (black, A, B), mGluR1-KD + mOrange PCs (orange, C), P/Q-KD + ZFP64-KD PCs (magenta, A), P/Q-KD + ZFP64-Res PCs (cyan, B), and mGluR1-KD + ZFP64-KD PCs (brown, C). Controls are against P/Q-KD + ZFP64-KD (A), P/Q-KD + ZFP64-Res (B), and mGluR1-KD + ZFP64-KD (C), respectively. Scale bars, 1 nA and 20 ms for A and B; 500 pA and 20 ms for C. The sample numbers of PCs are shown in parentheses. ∗∗∗*p* < 0.001, ∗∗*p* < 0.01, n.s. *p* > 0.05. Statistical tests and values are: (A) Kruskal-Wallis test, *H*_(2)_ = 17.5, *p* < 0.001, followed by Steel-Dwass test, *p* < 0.001 (Control vs. P/Q-KD + mOrange), *p* < 0.001 (Control vs. P/Q-KD + ZFP64-KD), and *p* = 0.965 (P/Q-KD + mOrange vs. P/Q-KD + ZFP64-KD); (B) Kruskal-Wallis test, *H*_(2)_ = 14.7, *p* < 0.001, followed by Steel-Dwass test, *p* = 0.0016 (Control vs. P/Q-KD + mOrange), *p* = 0.0017 (Control vs. P/Q-KD + ZFP64-Res), and *p* = 0.880 (P/Q-KD + mOrange vs. P/Q-KD + ZFP64-Res); (C) Kruskal-Wallis test, *H*_(2)_ = 44.9, *p* < 0.001, followed by Steel-Dwass test, *p* = 0.0032 (Control vs. mGluR1-KD + mOrange), *p* < 0.001 (Control vs. mGluR1-KD + ZFP64-KD), and *p* = 0.0036 (mGluR1-KD + mOrange vs. mGluR1-KD + ZFP64-KD).

We next sought to elucidate whether ZFP64 contributes to CF synapse elimination along the P/Q-VDCC- or mGluR1-mediated signaling pathway in PCs. To examine the possibility that ZFP64 functions downstream of P/Q-VDCC, we prepared mice with P/Q-VDCC-KD (P/Q-KD) in PCs and those with P/Q- and ZFP64-double KD in PCs and compared the degree of multiple CF innervation between them during P19 to P30. We found that the frequency distribution of PCs for the number of CFs was essentially identical between PCs with single P/Q-KD (P/Q-KD + mOr) and PCs with double P/Q- and ZFP64-KD (P/Q-KD + ZFP64-KD), indicating that the effect of ZFP64-KD on CF synapse elimination was occluded by P/Q-KD (Figure 2A). We then examined whether the expression of ZFP64-Res, which was used in Figure 1F, restored the impaired CF synapse elimination induced by P/Q-KD in PCs. However, we found no difference between PCs with P/Q-KD (P/Q-KD + mOr) and PCs with P/Q-KD and ZFP64-Res expression (P/Q-KD + ZFP64-Res) in the degree of multiple CF innervation (Figure 2B). This finding implicates that ZFP64 expression alone is insufficient to restore the impaired CF synapse elimination by the lack of P/Q-VDCC in PCs. Some factors produced by P/Q-VDCC may be required to act with ZFP64 to complete CF synapse elimination. Next, to test whether ZFP64 functions along the mGluR1-mediated pathway, we prepared mice with mGluR1-KD in PCs and those with mGluR1 and ZFP64-double KD in PCs. We found that the percentage of PCs innervated by more than two CFs was significantly higher in PCs with double mGluR1 and ZFP64-KD (mGluR1-KD + ZFP64-KD) than in PCs with single mGluR1-KD (mGluR1-KD + mOr) (Figure 2C), indicating that the effect of mGluR1-KD and that of ZFP64-KD are additive for CF synapse elimination. This result suggests that ZFP64 functions independently of mGluR1-mediated signaling cascades in PCs for CF synapse elimination.

However, these phenotypes of ZFP64-KD mice were milder than those of P/Q-VDCC-cKO^19^ and PCs with miRNA-induced P/Q-VDCC KD.^23^ To compare the effects between ZFP64-KD and P/Q-KD PCs, we performed P/Q-KD in PCs during postnatal development. We confirmed that the early phase of CF elimination was severely impaired in P/Q-KD PCs (Figures S3A and S3B), indicating that ZFP64-KD did not reproduce all the phenotypes of P/Q-KD in PCs.

### Transient impairments of the CF translocation and perisomatic CF terminal elimination in ZFP64-KD PCs

**Figure 3:**
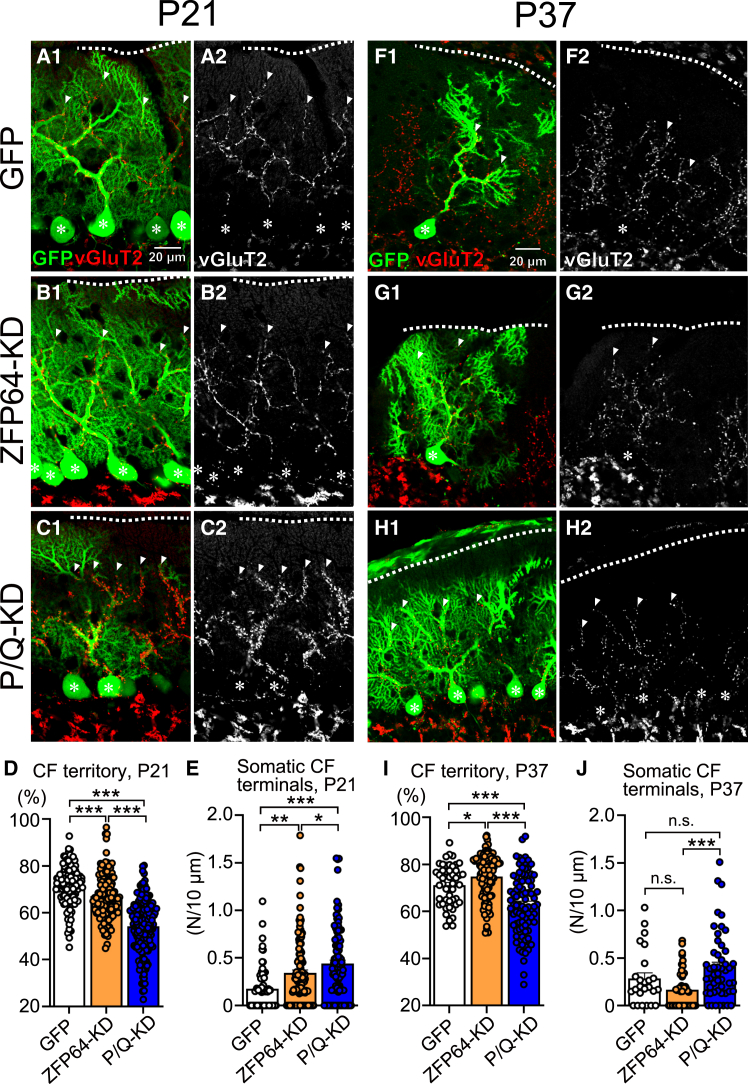
ZFP64-KD reduces CF translocation and increases surplus CF terminals on the PC soma at P21 but not at P37 (A–J) Double fluorescent labeling for GFP (green) and vGluT2 (red) of the cerebellum at P21 (A–C) and P37 (F–H) from GFP control mice (A, F), ZFP64-KD mice (B, G), and P/Q-KD mice (C and H). The dotted lines show the molecular layer boundary. Summary bar graphs for P21 (D, E) and P37 (I, J) represent the CF territory over the PC dendritic arbors (D and I, *n* = 127, 48 for control, *n* = 131, 111 for ZFP64-KD and *n* = 166, 86 for P/Q-KD) and the number of vGluT2 terminals per 10 μm along GFP-expressing PC soma (E and J, *n* = 76, 25 for control, *n* = 123, 52 for ZFP64-KD and *n* = 102, 50 for P/Q-KD). Data are represented as mean ± SEM. ∗∗∗*p* < 0.001, ∗∗*p* < 0.01, n.s. *p* > 0.05. Statistical tests and values are: (D) Kruskal-Wallis test, *H*_(2)_ = 158.2, *p* < 0.001, followed by Steel-Dwass test, *p* < 0.001 (Control vs. ZFP64-KD), *p* < 0.001 (Control vs. P/Q-KD), and *p* < 0.001 (ZFP64-KD vs. P/Q-KD); (E) Kruskal-Wallis test, *H*_(2)_ = 28.4, *p* < 0.001, followed by Steel-Dwass test, *p* = 0.002 (Control vs. ZFP64-KD), *p* < 0.001 (Control vs. P/Q-KD), and *p* = 0.044 (ZFP64-KD vs. P/Q-KD); (I) Kruskal-Wallis test, *H*_(2)_ = 44.5, *p* < 0.001, followed by Steel-Dwass test, *p* = 0.027 (Control vs. ZFP64-KD), *p* < 0.001 (Control vs. P/Q-KD), and *p* < 0.001 (ZFP64-KD vs. P/Q-KD); (J) Kruskal-Wallis test, *H*_(2)_ = 14.8, *p* < 0.001, followed by Steel-Dwass test, *p* = 0.211 (Control vs. ZFP64-KD), *p* = 0.302 (Control vs. P/Q-KD), and *p* < 0.001 (ZFP64-KD vs. P/Q-KD).

After a single strong CF is selected in each PC by P7, the strongest CF extends its innervation along PC dendrites from around P9 (CF translocation), and in parallel, redundant CF terminals are eliminated from the PC soma.^6^^,^^29^ Since these events were severely impaired in P/Q-VDCC-cKO,^19^ we examined whether ZFP64-KD in PCs affected the CF translocation and the CF terminal elimination from the PC soma by comparing the CF synaptic territories over PC dendritic arbors and the densities of CF terminals on the PC soma among control, ZFP64-KD, and P/Q-KD PCs at P21 and P37 (Figure 3). We visualized CF terminals by immunohistochemical labeling using an antibody against the CF terminal marker vGluT2. We estimated the extent of CF translocation over the PC dendritic arbor by measuring the relative height of vGluT2-positive CF terminals to the thickness of the molecular layer that reflects the height of PC dendritic arbors (Figures 3A–3D and 3F–3I). Moreover, we measured the density of vGluT2-positive CF terminals on the EGFP-positive PC soma (Figures 3A–3C, 3E–3H, and 3J). At P21, the degree of CF translocation was significantly lower in both ZFP64 and P/Q-KD PCs than in control PCs (Figures 3A–3C, and 3D). The reduction was much milder in ZFP64-KD PCs than in P/Q-KD PCs compared to control PCs (Figure 3D). The density of perisomatic CF terminals was significantly higher in ZFP64-KD and P/Q-KD PCs than in control PCs (Figure 3E). Moreover, anterograde labeling of a subset of CFs with a fluorescent tracer DTR-594 injected into the inferior olive showed that in both ZFP64-KD and P/Q-KD mice the PC soma at P21 was enwrapped by DTR-negative but vGluT2-positive CF terminals together with DTR- and vGluT2-double positive CF terminals (Figures S3C–S3H), indicating that such PCs were innervated by more than two CFs with different cellular origins in the inferior olive. However, at P37, the degree of CF translocation was slightly higher and the density of perisomatic CF terminals was at the same level in ZFP64-KD PCs as in control PCs (Figures 3F, 3G, 3I, and 3J). In contrast, the degree of CF translocation was significantly lower and the density of perisomatic CF terminals was at the same level in P/Q-KD PCs as in control PCs (Figures 3F, 3H, 3I, and 3J), showing that ZFP64-KD in PCs caused milder and transient effects than P/Q-KD in PCs. These results suggest that ZFP64 contributes to the translocation of the strongest CF to the PC dendrite and the removal of redundant CF synapses from the PC soma until around P21, but that the effects of the lack of ZFP64 do not persist into adolescence around P37.

### ZFP64-KD greatly enhances PF to PC excitatory transmission transiently during the 2^nd^ postnatal week

**Figure 4:**
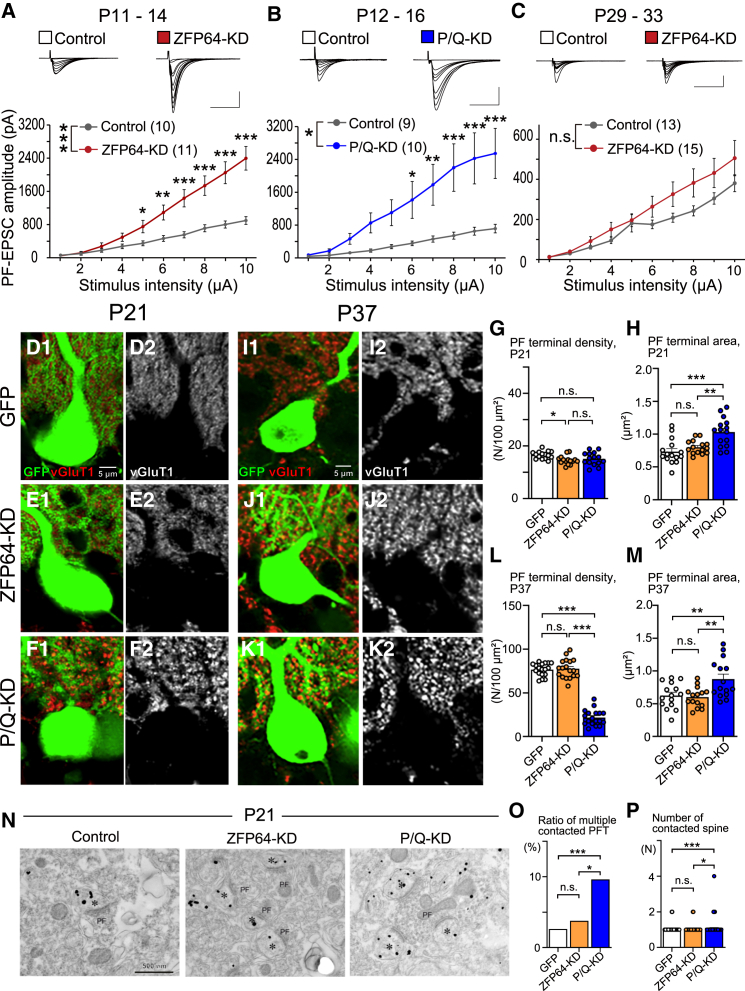
ZFP64-KD enhances PF to PC synaptic transmission transiently during postnatal development (A–C) (Upper panels) Representative traces of PF-EPSCs recorded from control GFP-negative PCs (left), GFP-positive ZFP64-KD PCs (right in A and C), and a P/Q-KD PC (right in B). The traces recorded at gradually decreasing stimulus intensities from 10 to 1 μA are superimposed. The holding potential was −70 mV. Scale bar: 1 nA (A and B), 0.2 nA (C), and 20 ms. (Lower panels) Summary graphs showing the stimulus-response relationships of PF-EPSCs for control (black) and ZFP64-KD (red) PCs during P11-14 (A), for control (black) and P/Q-KD (blue) PCs during P12-16 (B), and for control (black) and ZFP64-KD (red) PCs during P29-33 (C). The sample numbers of PCs are shown in parentheses. Statistical tests and values are: (A) Two-way repeated measures (rm)ANOVA, *F*_(9, 171)_ = 78.9, *p* < 0.001 (stimulus), *F*_(1, 19)_ = 14.3, *p* < 0.001 (KD), *F*_(9, 171)_ = 18.1, *p* < 0.001 (stimulus x KD), followed by Tukey’s test, *p* = 0.954 (1 μA), *p* = 0.940 (2 μA), *p* = 0.633 (3 μA), *p* = 0.279 (4 μA), *p* = 0.045 (5 μA), *p* = 0.002 (6 μA), and *p* < 0.001 (7–10 μA) (Control vs. ZFP64-KD); (B) Two-way rmANOVA, *F*_(9, 153)_ = 17.3, *p* < 0.001 (stimulus), *F*_(1, 17)_ = 7.3, *p* = 0.015 (KD), *F*_(9, 153)_ = 5.9, *p* < 0.001 (stimulus x KD), followed by Tukey’s test, *p* = 0.942 (1 μA), *p* = 0.811 (2 μA), *p* = 0.445 (3 μA), *p* = 0.131 (4 μA), *p* = 0.064 (5 μA), *p* = 0.018 (6 μA), *p* = 0.0033 (7 μA), and *p* < 0.001 (8–10 μA) (Control vs. P/Q-KD); (C) Two-way rmANOVA, *F*_(9, 234)_ = 53.1, *p* < 0.001 (stimulus), *F*_(1, 26)_ = 2.0, *p* = 0.174 (KD), *F*_(9, 234)_ = 1.9, *p* = 0.051 (stimulus x KD). (D–M) Double fluorescent labeling for GFP (green) and vGluT1 (red) of the cerebellum at P21 (D–F) and P37 (I–K) from a GFP control mouse (D and I), a ZFP64-KD mouse (E and J), and a P/Q-KD mouse (F and K). The summary bar graphs show the density of PF terminals per 100 μm^2^ at P21 (G, *n* = 15) and P37 (L, *n* = 18), and the PF terminal area at P21 (H, *n* = 15) and P37 (M, *n* = 15). Statistical tests and values are: (G) One-way ANOVA, *F*_(2, 42)_ = 4.4, *p* = 0.018, followed by Tukey’s test, *p* = 0.017 (Control vs. ZFP64-KD), *p* = 0.100 (Control vs. P/Q-KD), and *p* = 0.722 (ZFP64-KD vs. P/Q-KD); (H) One-way ANOVA, *F*_(2, 42)_ = 11.8, *p* < 0.001, followed by Tukey’s test, *p* = 0.529 (Control vs. ZFP64-KD), *p* < 0.001 (Control vs. P/Q-KD), and *p* = 0.0026 (ZFP64-KD vs. P/Q-KD); (L) One-way ANOVA, *F*_(2, 51)_ = 223, *p* < 0.001, followed by Tukey’s test, *p* = 0.952 (Control vs. ZFP64-KD), *p* < 0.001 (Control vs. P/Q-KD), and *p* < 0.001 (ZFP64-KD vs. P/Q-KD); (M) One-way ANOVA, *F*_(2, 42)_ = 7.2, *p* = 0.002, followed by Tukey’s test, *p* = 0.952 (Control vs. ZFP64-KD), *p* = 0.0087 (Control vs. P/Q-KD), and *p* = 0.0038 (ZFP64-KD vs. P/Q-KD). (N) Images from pre-embedding immunoelectron microscopy for GFP (gold particle) and vGluT1 (DAB staining, PF). Asterisks show PC spines contacting PF terminals. (O and P) Summary bar graphs showing the ratio of multiple contacted PF terminals and the number of contacted PC spines per PF terminal (PFT). Statistical tests and values are: (O) Kruskal-Wallis test, *H*_(2)_ = 21.7, *p* < 0.001, followed by Steel-Dwass test, *p* = 0.641 (Control vs. ZFP64-KD), *p* < 0.001 (Control vs. P/Q-KD), and *p* = 0.011 (ZFP64-KD vs. P/Q-KD); (P) Kruskal-Wallis test, *H*_(2)_ = 21.7, *p* < 0.001, followed by Steel-Dwass test, *p* = 0.641 (Control vs. ZFP64-KD), *p* < 0.001 (Control vs. P/Q-KD), and *p* = 0.011 (ZFP64-KD vs. P/Q-KD). ∗∗∗*p* < 0.001, ∗∗*p* < 0.01, ∗*p* < 0.05, n.s. *p* > 0.05. Data are represented as mean ± SEM.

We then examined whether ZFP64-KD in PCs impacts PF to PC excitatory synaptic transmission. We compared the input-output relationship of PF-EPSCs between ZFP64-KD PCs and P/Q-KD PCs during the second postnatal week. We found that the amplitudes of PF-EPSCs were larger in ZFP64-KD than in control PCs at PF stimulus intensity from 5 to 10 μA during P11-14 (Figure 4A). Similarly, the amplitudes of PF-EPSCs were larger in P/Q-KD PCs than in control PCs at PF stimulus intensity from 6 to 10 μA during P12-16 (Figure 4B). In addition, the paired-pulse ratio (PPR) of PF-EPSCs at 50 ms intervals was smaller in both ZFP64-KD and P/Q-KD PCs than in control PCs (Control for ZFP64-KD: 1.63 ± 0.06, ZFP64-KD: 1.32 ± 0.03; Control for P/Q-KD: 1.74 ± 0.05 P/Q-KD: 1.30 ± 0.06, both *p* < 0.001, Student’s *t* test), indicating the enhanced glutamate release probability from PF terminals. These changes in PF-EPSCs are consistent with those observed in P/Q-VDCC-cKO mice aged from P14 to P21,^21^ indicating that PF to PC excitatory synaptic transmission was greatly enhanced similarly in ZFP64-KD and P/Q-KD PCs during around P11 to around P16. However, when we examined adolescent mice from P29 to P33, the input-output relation and PPR of PF-EPSCs showed no significant difference between control and ZFP64-KD PCs (Figure 4C, PPR, Control: 1.79 ± 0.06, ZFP64-KD: 1.68 ± 0.05, *p* = 0.208, Student’s *t* test). These results indicate that ZFP64-KD in PCs transiently enhanced PF to PC synapse transmission similarly to P/Q-KD in PCs, but the enhancements did not persist in adolescence.

GABAergic inhibition of PCs has been shown to regulate CF synapse elimination.^24^^,^^25^^,^^26^ We, therefore, checked whether GABAergic inhibition was altered by ZFP64-KD in PCs by recording miniature inhibitory postsynaptic currents (mIPSCs)^30^ in ZFP64-KD and control PCs (Figures S4A–S4C). We found no difference between ZFP64-KD and control PCs in either the frequency or the amplitude of mIPSCs (Figures S4B and S4C). These results indicate that the impairment of CF elimination by ZFP64-KD is not attributable to alterations in GABAergic inhibition to PCs.

### Minor changes in the PF synaptic territory and the PF terminal morphology by ZFP64-KD

PF to PC synapse formation greatly influences the establishment of CF to PC synaptic wiring during postnatal development.^31^^,^^32^^,^^33^ In PC-specific P/Q-VDCC-cKO mice, the PF innervation territory over the PC dendritic arbor expanded, and PF to PC excitatory synaptic transmission was greatly enhanced.^19^^,^^20^^,^^21^ We, therefore, investigated whether ZFP64-KD in PCs affected the morphology of PF synapses by immunohistochemical labeling of PF synaptic terminals using an antibody against the PF terminal marker vGluT1. In the molecular layer surrounding control PCs, punctate vGluT1 signals were evenly distributed at P21 and P37 (Figures 4D–4M). In contrast, at P21, ZFP64-KD PCs (Figure 4E), and P/Q-KD PCs (Figure 4F) were surrounded by relatively larger vGluT1 puncta than control PCs. We quantified the PF terminal density in the molecular layer and found a significant difference between control and ZFP64-KD PCs, but there was no significant difference between control and P/Q-KD PCs (Figure 4G). In contrast, the area of PF terminals measured as the area of vGluT1 puncta in the molecular layer was significantly larger for P/Q-KD PCs than for control PCs, whereas the value was not different between control and ZFP64-KD PCs (Figure 4H). At P37, the size of vGluT1 puncta, the PF terminal density, and the PF terminal area for ZFP64-KD PCs became similar to those for control PCs (Figures 4I, 4J, 4L, and 4M). In contrast, the PF terminal density was significantly lower, and the PF terminal area was larger for P/Q-KD PCs than for control PCs (Figures 4I and 4K–4M). It has been reported that almost all PF synapses in wild-type mice had one-to-one contact with PCs, whereas the larger PF terminals in P/Q-VDCC-cKO mice contacted multiple PC spines.^21^ Our immunoelectron microscopy analysis indicates that the ratio of the multiply contacted PF terminals in PCs and the number of contacted PC spines per PF terminal was significantly increased in P/Q-KD PCs but not in ZFP64-KD PCs compared to control PCs (Figures 4N–4P).

These results suggest that ZFP64-KD in PCs induced only minor, if any, alterations in the PF synaptic territory and the morphology of PF terminals compared to those by P/Q-KD in PCs.

### Altered gene expressions associated with synaptic function and development in ZFP64-KD PCs during postnatal development

**Figure 5:**
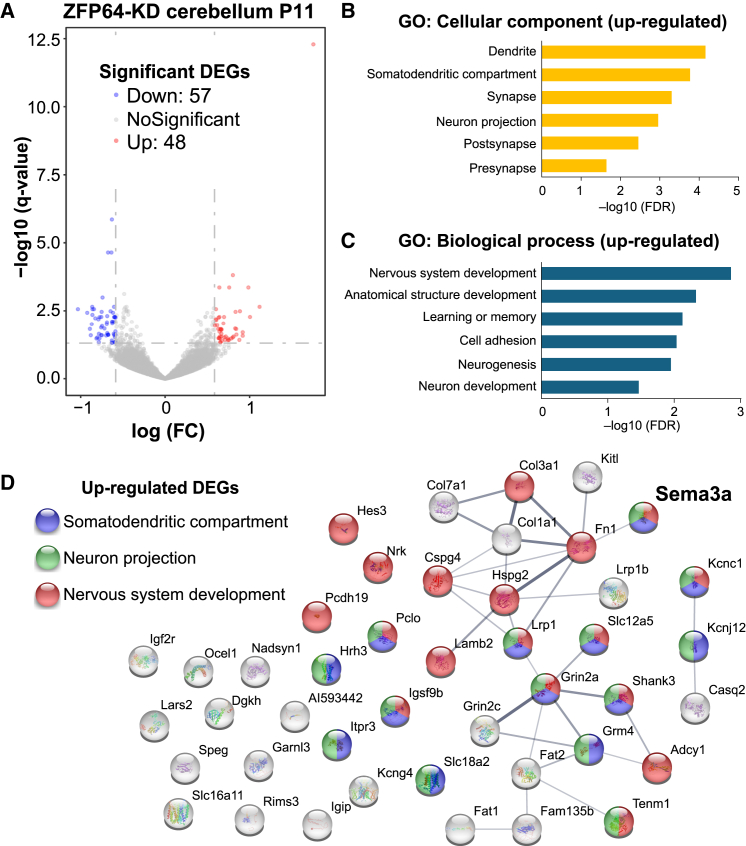
ZFP64-KD in PCs alters gene expression in the developing cerebellum (A) Volcano plot showing DEGs in the cerebellum at P11 between control mice expressing GFP in PCs and mice with ZFP64-KD in PCs (*n* = 4 samples for each group). Blue and red dots indicate 57 downregulated and 48 upregulated genes, respectively. The threshold of log (Fold change) is ±0.58 (0.67, 1.5 times changes against control), and that of *q* value is < 0.05. (B and C) Gene ontology (GO) annotations related to cellular component (B) and biological process (C) associated with upregulated DEGs. (D) Protein-protein interaction network analysis of upregulated DEGs among different aspects of synaptic function. *Sema3a* is known as a gene involved in CF synapse strengthening/maintenance in developing PCs (bold).

As a transcription regulator, ZFP64 has been previously reported to play crucial roles in various cellular functions.^27^^,^^28^ Therefore, it is expected that ZFP64 could orchestrate diverse molecular and cellular events by regulating the expression of its target genes in the developing cerebellum. To assess how ZFP64 contributes to expression patterns of transcriptomes, we conducted RNA-seq on samples from fluorescence-enriched regions of the cerebellar tissues dissected from ZFP64-KD mice and control mice with L7-GFP expression at P11 (Figure 5). We then identified differentially expressed genes (DEGs) in ZFP64-KD cerebellar samples (with >1.5 or <0.67 times changes compared to the expression levels in control samples), including 48 up-regulated and 57 downregulated genes (Figure 5A). The gene ontology (GO) analysis indicated that the upregulated DEGs were primarily enriched in cellular components relevant to “dendrite”, “somatodendritic compartment”, “synapse”, and “neuron projection” (Figure 5B). Concerning biological function, the upregulated DEGs were found to be involved in various neurodevelopment processes such as “nervous system development”, “anatomical structure development”, and “neuron development” (Figure 5C). Some of these proteins were predicted to interact with each other based on the protein-protein interaction analysis by the STRING program (Figure 5D). Among upregulated DEGs, we focused on *Sema3a* (Sema3A) (log(FC) = 0.70, 1.63 times against control, Figure 5D) because this molecule is strongly expressed in PCs and has been demonstrated to counteract CF synapse elimination by strengthening/maintaining CF synapses on PCs from around P8 to around P18.^16^ We tested whether Sema3A is involved in the impaired CF synapse elimination in ZFP64-KD PCs (see in the further section).

In contrast to upregulated DEGs, the GO analysis for the downregulated DEGs showed that they were predominantly associated with the annotation of “cell cycle”, and “cell division”, forming complex interactions among them (Figure S5A and S5B). The downregulated DEGs also included *Atoh1* and *Mfap4*, highly enriched in granule cell precursors/granule cells in the external granular layer,^34^^,^^35^ suggesting that the granule cell development might be indirectly affected in the PC-specific ZFP64-KD cerebellum at around P11. Although such downregulated DEGs might affect CF synapse elimination, we have not investigated their potential roles further in the present study.

### Sema3A is a potential target of ZFP64 for CF synapse elimination

**Figure 6:**
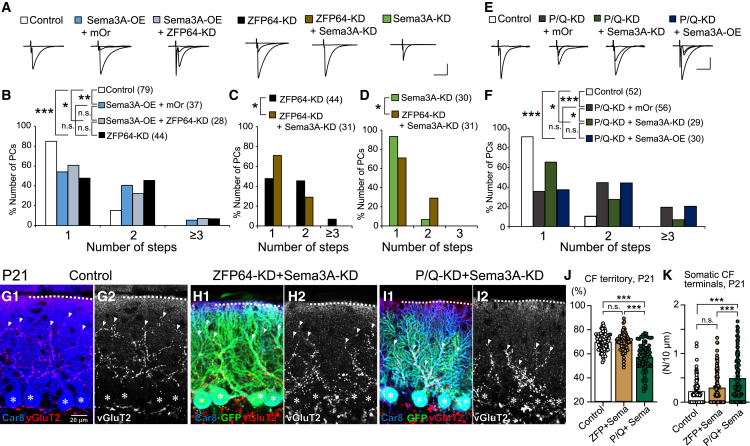
ZFP64 mediates CF synapse elimination partially by inhibiting Sema3A expression (A) Sample traces of CF-EPSCs recorded from an untransfected control (white), a Sema3A-OE + mOrange (blue), a Sema3A-OE + ZFP64-KD (blue gray), a ZFP64-KD (black), a Sema3A-KD + ZFP64-KD (brown), and a Sema3A-KD (green) PC. Scale bars, 1 nA and 20 ms. (B) Frequency distribution histograms showing the number of CFs innervating each PC during P19-30 for PCs of untransfected control, Sema3A-OE + mOrange, Sema3A-OE + ZFP64-KD, and ZFP64-KD. Statistical tests and values are: Kruskal-Wallis test, *H*_(3)_ = 22.5, *p* < 0.001, followed by Steel-Dwass test, *p* = 0.0016 (Control vs. Sema3A-OE + mOrange), *p* < 0.001 (Control vs. ZFP64-KD), *p* = 0.029 (Control vs. Sema3A-OE + ZFP64-KD), *p* = 0.973 (Sema3A-OE + mOrange vs. Sema3A-OE + ZFP64-KD), *p* = 0.939 (Sema3A-OE + mOrange vs. ZFP64-KD), and *p* = 0.774 (Sema3A-OE + ZFP64-KD vs. ZFP64-KD). (C and D) Frequency distribution histograms showing the number of CFs innervating each PC during P19-30 for PCs of ZFP64-KD (black) and ZFP64-KD + Sema3A-KD (brown) (C) and for PCs of Sema3A-KD (green) and ZFP64-KD + Sema3A-KD (brown) (D). Statistical tests and values are: Mann-Whitney *U* test, (C) *p* = 0.024 (ZFP64-KD vs. ZFP64-KD + Sema3A-KD) and (D) *p* = 0.012 (Sema3A-KD vs. ZFP64-KD + Sema3A-KD). The data of ZFP64-KD are the same as those in B. (E and F) Sample traces of CF-EPSCs (E) and frequency distribution histograms showing the number of CFs innervating each PC during P19 to P30 (F) for PCs of untransfected control (white), P/Q-KD + mOrange (gray), P/Q-KD + Sema3A-KD (dark green), and P/Q-KD + Sema3A-OE (dark blue). Scale bars, 1 nA and 20 ms. Statistical tests and values are: Kruskal-Wallis test, *H*_(3)_ = 40.8, *p* < 0.001, followed by Steel-Dwass test, *p* < 0.001 (Control vs. P/Q-KD + mOrange), *p* = 0.025 (Control vs. P/Q-KD + Sema3A-KD), *p* < 0.001 (Control vs. P/Q-KD + Sema3A-OE), *p* = 0.041 (P/Q-KD + mOrange vs. P/Q-KD + Sema3A-KD), *p* = 0.999 (P/Q-KD + mOrange vs. P/Q-KD + Sema3A-OE), and *p* = 0.101 (P/Q-KD + Sema3A-KD vs. P/Q-KD + Sema3A-OE). (G–I) Triple immunofluorescent labeling for Car8 (blue), GFP (green), and vGluT2 (red or white) in a GFP-negative untransfected control region (G), a region of ZFP64-KD + Sema3A-KD (H), and that of P/Q-KD + Sema3A-KD (I) of the cerebellum of mice at P21 transfected with miRNAs for ZFP64-KD + Sema3A-KD or P/Q-KD + Sema3A-KD at P1-2. (J and K) Summary bar graphs show the CF territory over the PC dendritic arbors (J, *n* = 80, Control; *n* = 84, ZFP64-KD + Sema3A-KD; *n* = 83, P/Q-KD + Sema3A-KD; Statistical tests and values are: Kruskal-Wallis test, *H*_(2)_ = 63.3, *p* < 0.001, followed by Steel-Dwass test, *p* = 0.467 (Control vs. ZFP64-KD + Sema3A-KD), *p* < 0.001 (Control vs. P/Q-KD + Sema3A-KD), and *p* < 0.001 (ZFP64-KD + Sema3A-KD vs. P/Q-KD + Sema3A-KD)) and the number of vGluT2 terminals per 10 μm along the PC soma (K, *n* = 106, Control; *n* = 125, ZFP64-KD + Sema3A-KD; *n* = 108, P/Q-KD + Sema3A-KD; Kruskal-Wallis test, *H*_(2)_ = 20.5, *p* < 0.001, followed by Steel-Dwass test, *p* = 0.502 (Control vs. ZFP64-KD + Sema3A-KD), *p* < 0.001 (Control vs. P/Q-KD + Sema3A-KD), and *p* < 0.001 (ZFP64-KD + Sema3A-KD vs. P/Q-KD + Sema3A-KD)). ∗∗∗*p* < 0.001, ∗∗*p* < 0.01, ∗*p* < 0.05, n.s. *p* > 0.05. Data are represented as mean ± SEM.

To investigate whether the upregulation of Sema3A in PCs affects CF synapse elimination, we overexpressed Sema3A and EGFP (Sema3A-OE) together with mOrange (mOr) in PCs by injecting lentivirus carrying the cDNAs for these molecules into the cerebellum at P1-2. We then examined CF innervation patterns by analyzing CF-EPSCs from P19 to P30. We found that the degree of multiple CF innervation was significantly higher in Sema3A-OE PCs than in control PCs (Figures 6A and 6B), suggesting that excess Sema3A in PCs impairs CF synapse elimination. Moreover, analogous to ZFP64-KD PCs, Sema3A-OE PCs showed a reduced dendritic translocation of CFs and an increased density of somatic CF terminals (Figures S6A–S6F). We then overexpressed Sema3A and knocked down ZFP64 in the same PCs and compared the effect of the combined Sema3A-OE and ZFP64-KD (Sema3A-OE + ZFP64-KD) with that of Sema3A-OE alone and ZFP64-KD alone (Figures 6A and 6B). We found no significant difference in the degree of multiple CF innervation among the three groups of PCs (Figure 6B), implying that the effects of Sema3A on CF synapse elimination were saturated in ZFP64-KD PCs. This result suggests that ZFP64-KD in PCs impairs CF synapse elimination through excess Sema3A expression in PCs. To check the validity of this possibility, we examined whether Sema3A-KD rescued the impaired CF synapse elimination in ZFP64-KD PCs. We confirmed the previous report that Sema3A-KD in PCs did not affect CF mono innervation from P19 to P30, although it accelerated CF synapse elimination from around P8 to around P18^16^ (Figures S7A and S7B). We found that the degree of multiple CF innervation in ZFP64 and Sema3A double-KD PCs (ZFP64-KD + Sema3A-KD) was significantly lower than in ZFP64-KD PCs (Figures 6A and 6C) but higher than in Sema3A-KD PCs (Figures 6A and 6D), and was not significantly different from untransfected control PCs without EGFP expression (Figures S7C and S7D). These results indicate that Sema3A-KD partially rescued the impaired CF synapse elimination in ZFP64-KD in PCs and suggest that ZFP64 mediates CF synapse elimination partially by suppressing Sema3A expression in PCs.

Given that Sema3A-KD can partially restore the impaired CF synapse elimination by ZFP64-KD or P/Q-KD (Figures 6C and 6D), we explored the effects of Sema3A-KD on P/Q-KD in PCs. We found that the degree of multiple CF innervation was significantly lower in PCs with P/Q-VDCC and Sema3A double-KD (P/Q-KD + Sema3A-KD) than in PCs with single P/Q-KD (P/Q-KD + mOr) (Figures 6E and 6F), indicating that downregulation of Sema3A could also partially recover the impaired CF synapse elimination by P/Q-KD in PCs. In contrast, Sema3A-OE induced no effect on the degree of multiple CF innervation in P/Q-KD PCs (Figures 6E and 6F), suggesting that the effects of Sema3A on CF synapse elimination were saturated in P/Q-KD PCs. We then performed immunohistochemical analyses to examine whether the reduced CF translocation and the impaired removal of somatic CF terminals in ZFP64-KD or P/Q-KD PCs at P21 were restored by adding Sema3A-KD. We found no significant differences between control PCs and PCs with ZFP64 and Sema3A double-KD (ZFP64-KD + Sema3A-KD) in the degree of CF translocation (Figures 6G, 6H, and 6J) and the density of somatic CF terminals (Figures 6G, 6H, and 6K). However, the degree of CF translocation was significantly smaller (Figures 6G, 6I, and 6J) and the density of somatic CF terminals was higher (Figures 6G, 6I, and 6K) in P/Q-VDCC and Sema3A double-KD PCs (P/Q-KD + Sema3A-KD) than in control PCs. These results show that Sema3A-KD was sufficient for ZFP64-KD PCs but not for P/Q-KD PCs to restore the reduced CF translocation and the impaired removal of somatic CF terminals at P21. Thus, other factors in addition to ZFP64 appear to be required for restoring the defects of P/Q-KD in PCs. Taken together, the results of the present study collectively suggest that ZFP64 acts presumably downstream of P/Q-VDCC, suppresses the expression of Sema3A, and facilitates the elimination of perisomatic CF synapses and the dendritic translocation of CFs.

## Discussion

We have disclosed that the transcription factor ZFP64, particularly the isoform 1, is expressed in PCs throughout development (Table S1) and is required mainly for the late phase of CF synapse elimination after P12 (Figure 1) (See limitations of the study). ZFP64 is considered to function presumably downstream of P/Q-VDCC (Figure 2). ZFP64-KD in PCs diminished the dendritic translocation of the strongest CF with excess CF terminals around PC somata at P21 (Figure 3). Conversely, ZFP64-KD in PCs enhanced PF to PC synaptic transmission around P12 (Figure 4). Notably, these changes in CF and PF synapses induced by ZFP64-KD in PCs were observed until around P30 but were not seen in adulthood (Figures 1, 2, 3, and 4). Moreover, the effects of ZFP64-KD in PCs were milder than those induced by the deletion of P/Q-VDCC in PCs, suggesting that ZFP64 mediates a part of the signaling pathway downstream of P/Q-VDCCs. We showed that Sema3A was upregulated in the cerebellum of PC-specific ZFP64-KD at P11 (Figure 5), and Sema3A-KD in PCs could ameliorate the impaired CF synapse elimination induced by ZFP64- or P/Q-KD in PCs (Figure 6). These findings collectively suggest that ZFP64 promotes CF synapse elimination partly by suppressing Sema3A expression during postnatal development. Thus, this study revealed the role of the transcription factor ZFP64 in the remodeling of neuronal wiring in the developing brain.

The developmental trajectory of CF innervation in ZFP64-KD PCs demonstrates that ZFP64 does not play a significant role in the early phase of CF elimination (Figure 1). Moreover, since ZFP64-KD PCs showed a normal disparity between strong and weak CFs (Table S3), ZFP64 does not play a crucial role in the selective strengthening of a single CF. Whereas CF-EPSC amplitudes were unaffected by ZFP64-KD in PCs, Sema3A-KD in PCs is reported to reduce CF-EPSC amplitudes.^16^ These results suggest that the downregulation of Sema3A by ZFP64 in PCs during normal postnatal development might reduce CF-EPSCs arising especially from weak CFs, thereby facilitating their elimination. A previous study showed that KD of the immediate-early gene *Arc* in PCs impairs the late phase of CF elimination through operating downstream of P/Q-VDCC.^23^ Arc may reduce the CF-EPSC amplitude by mediating AMPA receptor endocytosis^22^^,^^36^^,^^37^ in PCs, whereas Sema3A may promote AMPA receptor trafficking to distal dendrites and the neuronal surface^38^^,^^39^ of PCs. Thus, it is conceivable that Arc upregulation by P/Q-VDCC-mediated calcium rise and Sema3A downregulation by ZFP64 may collaboratively mediate the late phase of CF elimination along the P/Q-VDCC pathway.

We found that Sema3A-OE caused no additional effect and Sema3A-KD partially rescued the impaired CF synapse elimination in PCs with ZFP64-KD or P/Q-KD (Figure 6), suggesting that excess Sema3A action to maintain redundant CF synapses might result in a higher degree of multiple CF innervation in PCs lacking ZFP64 or P/Q-VDCC. A marked elevation of Sema3A expression in the cerebellar tissues with PC-specific ZFP64-KD at P11 (Figure 5) supports the above notion. Since previous studies showed that calcium rises through channels different from P/Q-VDCCs are required for Sema3A signaling in neurons,^40^^,^^41^ the action of Sema3A might be supported by intracellular calcium rises independent of P/Q-VDCCs in P/Q-KD PCs. In contrast, ZFP64-OE in P/Q-KD PCs could not restore the impaired CF synapse elimination (Figure 2B), suggesting that calcium rises or some other factors dependent on P/Q-VDCCs are required for the action of ZFP64. Previous studies have shown that T-type Ca^2+^ channels in PCs are functionally coupled to mGluR1^42^ and are activated during CF input.^43^^,^^44^ Although it remains unclear whether the T-type Ca^2+^ channels in PCs contribute to CF synapse elimination due to a lack of direct evidence the Ca^2+^ entry through not only P/Q- but also T-type-VDCCs could mediate the activity-dependent transcription regulation.^45^ Besides, a recent study showed that neural activity recruited ribosomes to the coding sequence and its upstream of ZFP64 transcripts in culture neurons and enhanced translation in dendrites.^46^ This raises the possibility that ZFP64 translation might be regulated by neural activity with Ca^2+^ signaling. We assume that with normal Ca^2+^ entry by PC activity, ZFP64 protein expression or activity is elevated by P11 and, consequently, Sema3A expression is downregulated, which permits the elimination of loser CF synapses from PC somata.

CF synapse elimination involves not only homosynaptic competition among multiply innervating CFs but also heterosynaptic competition between CFs and PFs.^31^^,^^32^^,^^33^ Moreover, proper strength of GABAergic inhibition to PCs is a prerequisite for CF synapse elimination.^24^^,^^25^^,^^26^ In ZFP64-KD PCs, we observed enhanced PF synaptic transmission (Figure 4), while GABAergic inhibition remained unaltered (Figure S4). The stronger PF synaptic inputs and the reduced dendritic translocation of CFs suggest that PFs may extend their innervation territory to more proximal dendrites of PCs. These phenotypes, while coherent with, but less severe than, those of P/Q-KD PCs (Figure 3), leave the question of whether the enhanced PF synaptic strength is primarily caused by the absence of ZFP64 or P/Q-VDCC, or a secondary effect due to the regressed CF innervation. Since Sema3A-KD in PCs is reported to enhance the amplitude of PF-EPSCs^16^ and the present study shows the upregulation of Sema3A function in ZFP64- or P/Q-KD PCs, the marked enhancement of PF to PC transmission in ZFP64-KD PCs (Figure 4) cannot be explained by the change in Sema3A expression in PCs. Factors other than Sema3A downstream of P/Q-VDCCs and ZFP64 may regulate the strength of PF to PC synaptic transmission directly or indirectly by controlling CF to PC synaptic transmission. Moreover, since Sema3A-KD was insufficient to recover the impaired CF synapse elimination in ZFP64- or P/Q-KD PCs completely (Figure 6), some unidentified factors must exist that function downstream of P/Q-VDCCs and ZFP64 and contribute to CF to PC and PF to PC synaptic wiring.

In addition to Sema3A, we identified several DEGs potentially involved in synaptic wiring. For example, *Fat2*, a possible GluD2-interacting protein^47^ and highly expressed in developing granule cells^48^ was included in upregulated DEGs (Figure 5). The upregulated *Fat2* might induce the excess formation of PF to PC synapses and contribute to enhance PF to PC excitatory synaptic transmission. We also found *Grin2a* and *Grin2c* encoding NMDA receptors and *Shank3* in upregulated DEGs. Since these genes are expressed in granule cells, NMDA receptor-mediated mossy fiber to granule cell excitatory synaptic transmission would be enhanced in the cerebellum of PC-specific ZFP64-KD. Since these upregulated genes function in cells other than PCs, the ZFP64-KD in PCs must have upregulated their expression indirectly or through a leaky infection of ZFP64-KD lentivirus to GCs (See limitations of the study). It remains to be determined if other DEGs altered in PC-specific ZFP64-KD cerebellum are involved in synaptic wiring and function in the developing cerebellum.

ZFP64 has 4 isoforms (isoform A to D) in humans, whereas the murine counterpart has 3 isoforms. Our expression analysis suggests the dominant expression of the isoform 1 in PCs (Table S1). Murine ZFP64 isoform 1 shares sequence similarities with human ZFP64 isoform A, but minor differences with human ZFP64 isoform B and C.^27^ The human ZFP64 isoform A is known to maintain the expression of KMT2A/MLL encoding a histone-lysine N-methyltransferase and is involved in leukemia growth via a C-terminal transactivation domain.^28^ Both isoforms 1 and 2 interact with Notch intracellular domain via their C-terminal region,^27^ suggesting a common function of ZFP64 through the C-terminal region. However, in our RNA-seq data, KMT2A- and Notch-related signaling molecules were not identified in the DEGs at P11 (Figures 5 and S5). Thus, these molecules do not seem to be regulated by ZFP64 at least in the developing cerebellum. Further investigation is needed to elucidate how ZFP64 regulates gene expression for shaping CF and PF synaptic wiring in developing PCs.

### Limitations of the study

The present study has several limitations that should be considered when interpreting the results. First, our KD experiments relied on the efficiency and stochastic nature of lentivirus infection in PCs and those of electrophysiological data sampling from PCs. These factors may have led to variability in phenotypes, making it difficult to detect statistically significant differences in the degree of multiple CF innervation between groups from P8 to P11 (Figure 1). Therefore, our results do not rule out a potential contribution of ZFP64 in the early phase of CF elimination, although the differences between the groups did not reach statistical significance. Second, although rare, some GCs were non-specifically infected by the lentivirus (Figure S2A). Such a leaky infection may have influenced the observed changes in gene expression in GCs detected by RNA-seq (Figure 5). Third, due to the lack of specific antibodies against ZFP64 and Sema3A for immunohistochemistry, we could not directly show the expression level of ZFP64 protein in P/Q-KD PCs or changes in the level of Sema3A protein in ZFP64-KD or P/Q-KD PCs. Therefore, how ZFP64 and Sema3A are regulated downstream of P/Q-VDCC remains unclear. Fourth, although our RNA-seq data suggest Sema3A is a potential target of ZFP64, whether and how the Sema3A expression is regulated by ZFP64 remains unknown.

## Resource availability

### Lead contact

Requests for further information and resources should be directed to and will be fulfilled by the lead contact, Masanobu Kano (mkano-tky@m.u-tokyo.ac.jp).

### Materials availability

This study did not generate new unique materials and reagents.

### Data and code availability

• The processed RNA-seq data used in Figure 5 have been deposited in DDBJ (https://www.ddbj.nig.ac.jp) as accession number E-GEAD-1055 and are publicly available as of the date of publication.
• This paper does not report original code.
• Any additional information required to reanalyze the data reported in this paper is available from the lead contact upon request.

## Acknowledgments

We appreciate Dr. A. Nienhuis for the gifts of the lentiviral backbone vector and the packaging plasmid, and Dr. H. Hirai for the Purkinje cell-tropic viral vector. We thank Drs. T.H. Kao, E. Lai, M.J. Choo, C.L. Mercier, K. Nagahama, T. Akamatsu, H. Suzuki, and H.C. Lin for helpful advice and discussions. We appreciate M. Sekiguchi, M. Watanabe-Suzuki, and T. Tanaka for technical assistance and K. Akasaka, K. Aoyama, M. Yoshino, and T. Hatada for animal care. This work was supported by Grants-in-Aid for Scientific Research (18H04012, 20H05915, 21H04785 to M.K.; JP22H04926, “Advanced Bioimaging Support”) from the 10.13039/501100001691Japan Society for the Promotion of Science (10.13039/501100001691JSPS) and the scholarship for visiting research student from the 10.13039/100008732Uehara Memorial Foundation to J.Z. Cartoons were created using the BioRender online software.

## Author contributions

J.Z., T.W., N.U., and M.K. designed the study and wrote the paper. J.Z. performed all the experiments and analyzed the data except for the following experiments. N.U. performed microarray analysis. T.W., Y.O., T.N., and K.M. performed a part of the electrophysiological experiments. T.W. analyzed RNA-seq data. K.K. performed fluorescent *in situ* hybridization. T.W., T.M., M.Y., and M.W. performed the immunohistochemistry experiments.

## Declaration of interests

The authors declare no competing interests.

## STAR★Methods

### Key resources table

**Table:** 

REAGENT or RESOURCE	SOURCE	IDENTIFIER
**Antibodies**		

Guinea pig anti-Car8	Nittobo	Cat# MSFR100510; RRID:AB_2571669
Rabbit anti-GFP	Nittobo	Cat# MSFR101890; RRID:AB_2491093
Rat anti-GFP	Nacalai Tesque	Cat# 04404-84; RRID:AB_10013361
Rabbit anti-vGluT1	Nittobo	Cat# MSFR106180; RRID:AB_2571616
Guinea pig anti-vGluT1	Nittobo	Cat# MSFR106220; RRID:AB_2571534
Goat anti-vGluT2	Nittobo	Cat# MSFR106260; RRID:AB_2571620

**Bacterial and virus strains**		

psPAX2	gift from Didier Trono	Addgene; plasmid# 15246
pCAG-VSVG	gift from Arthur Nien- huis	Addgene; plasmid# 35616

**Biological samples**		

HEK293T cell line	ATCC	CAT# CRL-3216; RRID: CVCL_0063

**Chemicals, peptides, and recombinant proteins**		

Picrotoxin	Tocris	CAT# 1128
tetrodotoxin	Nacalai Tesque	CAT# 32775-51
NBQX	Tocris	CAT# 0373
D-AP5	Tocris	CAT# 0106

**Deposited data**		

RNA-seq (processed data)	This paper	DDBJ: E-GEAD-1055

**Experimental models: Organisms/strains**		

Mouse:C57BL/6N	Japan SLC	RRID:MGI:2159965

**Oligonucleotides**		

ZFP64-KD1 5′- TGCTGCTGAAGTGCTGCTTACAGAGGG TTTTGGCCACTGACTGACCCTCTGTACAGCACTTCAG-3′	This paper	N/A
ZFP64-KD1 5′- CCTGCTGAAGTGCTGTACAGAGGGTCAG TCAGTGGCCAAAACCCTCTGTAAGCAGCACTTCAGC-3′	This paper	N/A
ZFP64-KD2 5′- TGCTGTGAACTTGGCACTACAGAGCCG TTTTGGCCACTGACTGACGGCTCTGTTGCCAAGTTCA-3′	This paper	N/A
ZFP64-KD2 5′- CCTGTGAACTTGGCAACAGAGCCGTCAG TCAGTGGCCAAAACGGCTCTGTAGTGCCAAGTTCAC-3′	This paper	N/A
P/Q-VDCC-KD 5′-TGCTGTTCCAATGAAGTATGGTTCCGG TTTTGGCCACTGACTGACCGGAACCACTTCATTGGAA-3′	Uesaka et al.^16^	N/A
P/Q-VDCC-KD 5′-CCTGTTCCAATGAAGTGGTTCCGGTCA GTCAGTGGCCAAAACCGGAACCATACTTCATTGGAAC-3′	Uesaka et al.^16^	N/A
mGluR1-KD 5′-TGCTGAAATCAGGGAGTCTCTGATGAG TTTTGGCCACTGACTGACTCATCAGACTCCCTGATTT-3′	Uesaka et al.^16^	N/A
mGluR1-KD 5′-CCTGAAATCAGGGAGTCTGATGAGTCA GTCAGTGGCCAAAACTCATCAGAGACTCCCTGATTTC-3′	Uesaka et al.^16^	N/A
Sema3A-KD 5′-TGCTGTATAGGTGCAGATTGGATGGAG TTTTGGCCACTGACTGACTCCATCCACTGCACCTATA-3′	Uesaka et al.^16^	N/A
Sema3A-KD 5′-CCTGTATAGGTGCAGTGGATGGAGTCAG TCAGTGGCCAAAACTCCATCCAATCTGCACCTATAC-3′	Uesaka et al.^16^	N/A
ZFP64-KD1-resistant form 5′-CCACAAAGGCATCCAGATTGCTA AAATGTTGTTTGCACAGTCCGCAGATGTGGATGTCCGGTG-3	This paper	N/A
ZFP64-KD1-resistant form 5′-CACCGGACATCCACATCTGCGG ACTGTGCAAACAACATTTTAGCAATCTGGATGCCTTTGTGG-3′	This paper	N/A
ZFP64-KD2-resistant form 5′-TTTCAAGTCCGAGCTGATTTT AAATTTAGCGCTGCACAGCCAGCACTGGAAGGGGGCG-3′	This paper	N/A
ZFP64-KD2-resistant form 5′-CGCCCCCTTCCAGTGCTGG CTGTGCAGCGCTAAATTTAAAATCAGCTCGGACTTGAAA-3′	This paper	N/A

**Recombinant DNA**		

pCL20c-trL7	Sawada et al.^49^	N/A

**Software and algorithms**		

PATCHMASTER	HEKA	RRID:SCR_000034
FITMASTER	HEKA	RRID:SCR_016233
SutterPatch	Sutter instrument	N/A
STRING	STRING	RRID:SCR_005223
Fiji (ImageJ)	NIH	RRID:SCR_002285
MetaMorph	Molecular Devices	RRID:SCR_002368
GraphPad Prism	GraphPad Software Inc	RRID:SCR_002798
EZR	Kanda^50^	https://www.jichi.ac.jp/saitama-sct/SaitamaHP.files/statmedEN.html
BioRender	BioRender	RRID:SCR_018361

### Experimental model and study participant details

C57BL/6N mice of both sexes randomly chosen at postnatal day 1 (P1) to P70 were used. Newborn pups were obtained from pregnant female mice purchased from the supplier (SLC, Japan). After viral injections to the cerebella of newborn mice at P1 to 2, they were returned to the mother mouse in a home cage (22–23°C, humidity of 30–60%) with a 12-h light/12-h dark cycle. The L7-GFP transgenic mice were used for the microarray analysis as reported previously.^16^ Sex differences were not analyzed in this study. All the experiments were conducted following the guidelines established by the animal welfare committees of the University of Tokyo, Hokkaido University, and the Japan Neuroscience Society.

### Method details

#### Viral vector constructs

Viral vectors were constructed as reported previously.^51^ Vesicular stomatitis virus G (VSV-G) pseudotyped lentiviral vectors (pCL20c)^52^ were used for transferring different plasmids into mice. The vectors were designed to express an enhanced green fluorescent protein (eGFP) or mOrange2 (mOrange) together with a microRNA (miRNA) and/or a cDNA under the control of a truncated L7 promoter (pCL20c-trL7) to yield a Purkinje cell (PC)-specific expression.^49^ For the vector-based RNA interference (RNAi) analysis, the following miRNAs engineered by BLOCK-iT Pol II miR RNA expression vector Kit (Invitrogen) were used:

5′- TGCTGCTGAAGTGCTGCTTACAGAGGGTTTTGGCCACTGACTGACCCTCTGT

ACAGCACTTCAG-3′ and 5′- CCTGCTGAAGTGCTGTACAGAGGGTCAGTCAGTGGCCAAAACCCTCTGTAA

GCAGCACTTCAGC-3′ for miRNA-1 targeting on a shared part of ZFP64 isoform 1 and 2; 5′- TGCTGTGAACTTGGCACTACAGAGCCGTTTTGGCCACTGACTGACGGCTCT

GTTGCCAAGTTCA-3′ and 5′- CCTGTGAACTTGGCAACAGAGCCGTCAGTCAGTGGCCAAAACGGCTCTGTA

GTGCCAAGTTCAC-3′ for miRNA-2 targeting on ZFP64 isoform 1;

5′-GCTGTTCCAATGAAGTATGGTTCCGGTTTTGGCCACTGACTGACCGGAAC

CACTTCATTGGAA-3′ and 5′-CCTGTTCCAATGAAGTGGTTCCGGTCAGTCAGTGGCCAAAACCGGAACCAT

ACTTCATTGGAAC-3′ for P/Q-VDCC miRNA; 5′-TGCTGAAATCAGGGAGTCTCTGATGAGTTTTGGCCACTGACTGACTCATCAG

ACTCCCTGATTT-3′ and 5′-CCTGAAATCAGGGAGTCTGATGAGTCAGTCAGTGGCCAAAACTCATCAGA

GACTCCCTGATTTC-3′ for mGluR1 miRNA; 5′-TGCTGTATAGGTGCAGATTGGATGGAGTTTTGGCCACTGACTGACTCCATC

CACTGCACCTATA-3′ and 5′-CCTGTATAGGTGCAGTGGATGGAGTCAGTCAGTGGCCAAAACTCCATCCAA

TCTGCACCTATAC-3′ for Sema3A miRNA.

These oligonucleotides were then subcloned into the 5′- side of GFP or 3′ side of mOrange in the pCL20c-trL7 vector. Wild-type cDNA for murine ZFP64 isoform 1 (GeneBank accession number, NM009564) was produced by RT-PCR utilizing cDNA library from the P14 mouse cerebellum. The cDNA for Sema3A (GeneBank accession number, NM_009152) was obtained from the P10 mouse cerebellum.^16^ To generate an RNAi-resistant form of ZFP64 for the rescue of the RNAi-mediated knockdown (KD) of this gene, 6–7 DNA point mutations of the miRNA targeting region in ZFP64 cDNA were designed using the QuikChange Lightning site-directed mutagenesis kit (Agilent Technologies). Mutated ZFP64 cDNA coded the same amino acid sequence as its original but avoided being recognized by the miRNA. The following sequences were used to generate RNAi-resistant form of ZFP64:

5′-CCACAAAGGCATCCAGATTGCTAAAATGTTGTTTGCACAGTCCGCAGATG

TGGATGTCCGGTG-3′ and 5′-CACCGGACATCCACATCTGCGGACTGTGCAAACAACATTTTAGCAATCTG

GATGCCTTTGTGG-3′ for the ZFP64 miRNA-1-resistant form, and 5′-TTTCAAGTCCGAGCTGATTTTAAATTTAGCGCTGCACAGCCAGCACTGGAAG

GGGGCG-3′ and 5′-CGCCCCCTTCCAGTGCTGGCTGTGCAGCGCTAAATTTAAAATCAGCTCGGACTTGAAA-3′ for the ZFP64 miRNA-2-resistant form.

The ZFP64 cDNA and the ZFP64 miRNA-1&2-resistant form of ZFP64 cDNA isoform 1 (ZFP64-Res) were respectively linked in-frame to mOrange2 via a picornavirus “self-cleaving” P2A peptide sequence to enable efficient bicistronic expression, and then subcloned into pCL20c-trL7. All the constructs were verified by DNA sequencing before use.

#### Virus preparation

The virus vector was produced by co-transfecting sub-confluent HEK293T cells with a mixture of two packaging plasmids (7 μg of psPAX2 and 3.5μg of pCAG-VSV-G) and a lentivirus vector (10 μg of pCL20c-tr7-ZFP64-miRNA-GFP, pCL20c-tr7-P/Q-VDCC-miRNA-GFP or pCL20c-tr7-ZFP64-Res-mOrange) as described previously,^23^^,^^51^ using a calcium phosphate precipitation method.^53^ The cultured medium was replaced with a fresh medium 16–24 h after the transfection. Forty to 48 h after the transfection, the medium containing the virus vector particles was harvested and then filtered through 0.22 μm filter membranes. After the filtration, the medium was centrifuged at 27,000 for 90 min. The viruses from 3 dishes were suspended in 30 μL of PBS and stored at 4°C.

#### Lentivirus infection *in vivo*

Lentivirus injection was made into the cerebellum of newborn C57BL/6N mice at P1-2. A mouse was anesthetized by inhalation of 1–2.5% isoflurane and fixed to a head clamping device. The cranium over the cerebellar vermis was exposed by midline sagittal incision. A hole was drilled at the midline with the meninges punctured. A 33-gauge Hamilton syringe affixed to a micropump (UltramicroPump II, World Precision Instruments (WPI)) was filled with 3.5 μL of virus solution. The tip of the syringe was placed on the cerebellar vermis. The viral solution was injected at a rate of 200 nL/min regulated by the microprocessor-based controller (Micro 4; WPI). After the injection, the syringe was left for 2 min in place before being withdrawn. Then, the wound was sutured, and the mouse was returned to its home cage.

#### Evaluation of knockdown efficiency

At first, 30–40% confluent HEK293T cells in a 24-well dish were transfected with 0.5 μg/well of control or a miRNA vector (ZFP64 miRNA-1, ZFP64 miRNA-2) expressing GFP. Six hours later, a mixture of 0.375 μg/well of a control or a miRNA vector and 0.125 μg/well of a ZFP64 cDNA or a Rescue vector expressing mOrange was transfected into the same wells. Two days later, the cells were fixed with 4% Paraformaldehyde (PFA) in 0.1 M phosphate buffer (PB). The fluorescence intensity of each well was measured under a confocal laser-scanning microscope (FV1200, Evident).

#### Electrophysiology

C57BL/6N mice at P8 to P70 were deeply anesthetized with carbon dioxide and decapitated immediately. The cerebellum was removed from the whole brain with part of the brain stem and the cerebrum, and immersed into chilled (4°C or lower) artificial cerebrospinal fluid (ACSF) composed of 125 mM NaCl, 2.5 mM KCl, 1.25 mM NaH_2_PO_4_, 26 mM NaHCO_3_, 2 mM CaCl_2_, 1 mM MgSO_4_, and 20 mM glucose bubbled with 95% O_2_ and 5% CO_2_. Parasagittal cerebellar slices with 250 μm thickness were prepared with a vibratome slicer (VT1200s, Leica) and then transferred to a reservoir chamber bathed with the same solution at room temperature. After at least 30 min of incubation for recovery, a slice was placed in a recording chamber located on the stage of an Olympus BX51WI microscope. The recording chamber was continuously perfused with oxygenated ACSF at 30°C containing 0.1 mM picrotoxin to block inhibitory synaptic currents for recording climbing fiber (CF) and parallel fiber (PF)-mediated synaptic responses. Whole-cell patch-clamp recordings were made from fluorescent-protein positive or control (visually recognized) PCs under the fluorescence microscope. The resistance of patch pipettes was 1.8–2.8 MΩ after filled with an internal solution composed of 60 mM CsCl, 10 mM Cs D-Gluconate, 20 mM TEA-Cl, 20 mM BAPTA, 4 mM MgCl_2_,30 mM HEPES, 4 mM Na_2_-ATP, and 0.4 mM Na_2_-GTP (pH 7.3 adjusted with CsOH). The pipette access resistance was compensated by 70%. All the current recordings were conducted with an EPC10 patch-clamp amplifier (HEKA Elektronik) or a Double IPA amplifier (Sutter instrument). The signals were filtered at 2–3 kHz and digitized at 20 kHz. Online data acquisition was performed using PatchMaster (HEKA Elektronik) or SutterPatch software (Sutter instrument) while Offline data was analyzed by FitMaster (HEKA-Electronik) or SutterPatch softwares and the MiniAnalysis Program (Version 6.0.7, Synaptosoft). To induce CF-EPSCs, the location of a stimulating pipette filled with ACSF was systematically moved within the granular cell layer 20–100 μm away from the PC layer. Pairs of stimulus pulses of 0.1 ms duration at 50 ms intervals were delivered at a frequency of 0.2 Hz. At each location, the stimulus intensity was gradually increased from 0 μA to 100 μA. For recording CF-EPSCs, the holding potential was set to −10 mV. When a CF was stimulated, an EPSC with a discernible step showing depression to the second of the stimulus pair (paired-pulse depression) was elicited. The number of CFs innervating the PC under recording was estimated from the number of discrete CF-EPSC steps as described previously.^14^^,^^29^ Since CF-EPSCs display paired-pulse depression whereas PF-mediated EPSCs (PF-EPSCs) exhibit paired-pulse facilitation, the two types of EPSCs were readily distinguished.^54^ To stimulate PFs, a glass pipette filled with the ACSF was placed in the molecular layer at 50–100 μm from the PC soma. For recording PF-EPSCs, the holding potential was −70 mV. The input-output relationship of PF-mediated EPSCs was obtained by measuring the amplitude of PF-EPSCs while decreasing the stimulus intensity from 10 μA to 1 μA by 1 μA steps. Miniature IPSCs of PCs were recorded at a holding potential of −70 mV in the ACSF containing 0.5 μM tetrodotoxin, 10 μM NBQX, and 50 μM D-AP5 for blocking Nav channels, AMPA receptors, and NMDA receptors, respectively.^30^

#### Quantification of disparity among the amplitudes of multiple CF-EPSCs in individual PCs

Two parameters, the disparity ratio and the disparity index, were exploited to quantify the disparity among the amplitudes of multiple CF-EPSCs recorded in each PC.^29^ To calculate the disparity ratio and disparity index, we firstly measured the amplitudes of individual CF-EPSCs in a given multiply-innervated PC and then numbered them in the order of their amplitudes as A_1_ to A_N_ (*N* ≥ 2; N is the number of CFs innervating a given PC. A_N_ is assumed to represent the largest CF-EPSC). The formulas are indicated below.

Disparity ratio = $\frac{\left(\frac{A_{1}}{A_{N}}+\frac{A_{2}}{A_{N}}+\cdots +\frac{A_{N}}{A_{N}}\right)}{\left(N-1\right)}$

Disparity index = $\frac{S.D.}{M}$

*M* = $\sum \frac{A_{i}}{N}$ (*i* =1,2,…*N*; *N* ≥ 2)

*S.D*. = $\sqrt{\sum \frac{{\left(A_{i}-M\right)}^{2}}{N-1}}$

A_i_ is the amplitude of CF-EPSC recorded at the same holding potential.

The disparity ratio reflects the average of the inverse proportion of the EPSC amplitude for the strongest CF to that for every other weaker CF. Accordingly, the disparity ratio will be small if the differences between the largest and the other smaller CF-EPSCs are large. On the other hand, the disparity index is the coefficient of variation for the amplitudes of multiple CF-EPSCs of a given PC. Therefore, if the variability among the EPSC amplitudes is high, the value of the disparity index will be large.

#### Isolation of PCs using fluorescence-activated cell sorter (FACS)

The FACS experiments have been performed and described previously in Uesaka et al.^16^ In brief, the cerebellum was extracted from L7-GFP transgenic mice at P4, P6, P7, P9, P13 and P15. Cerebellar tissues were digested in a dissociation solution comprising 0.2 mg/mL DL-cysteine HCl, 0.2 mg/mL bovine serum albumin, 1 mM HEPES, 1.5 mM MgSO_4_, 5 mg/mL glucose (pH 7.4), and Ca^2+^-free Hank’s balanced salt solution (HBSS). The enzymatic reaction was stopped by using 5 U/mL DNase I. Tissues were tenderly triturated utilizing broad and fine-tipped pipettes. Dissociated cells were collected and re-suspended in Ca^2+^ and Mg^2+^-free PBS. The cells were filtered through a 40 μm nylon mesh and underwent the cell sorting process by a FACS EPICS ALTRA (Beckman Coulter, Inc., CA, USA). To separate PCs, cells were passed through a 70 μm nozzle at a rate of 10000–20000 events/s at the first sorting. At the second sorting, the speed was changed to 1000 events/s. The sort decision was based on measuring forward scatter and GFP fluorescence.

#### Microarray analysis

Total RNA was extracted from the isolated PCs of L7-GFP mice by FACS and used as a template in the GeneChip Mouse Genome 430 2.0 DNA microarray (Affymetrix, CA, USA). Affymetrix GCOS and Microarray Suite (MAS) 5.0 were employed for raw image file processing. The MAS 5.0 includes a detection algorithm that can generate a detection *p*-value taking advantage of probe pair (perfect match/mismatch) intensities. *p*-value is then applied to determine whether a transcript is present or absent. For the gene expression in PCs, intensity data of ZFP64 probes were normalized to the signal intensity of GAPDH.

#### Immunofluorescence

Mice were transcardially perfused with 4% paraformaldehyde (PFA) in 0.1 M phosphate buffer (PB, pH 7.2) under deep pentobarbital anesthesia (100 μg/g body weight, intraperitoneal injection). Parasagittal sections of the cerebellar vermis (50-100 μm-thick) were cut using a VT1000S or VT1200S microslicer (Leica Microsystems) and immunostained using the free-floating method in glass test tubes or a 24-well dish. Phosphate-buffered saline (PBS, pH 7.4) containing 0.1% Triton X-100 (PBS-T) was used for incubation and washing. After blocking with 10% normal donkey serum (Jackson ImmunoResearch) for 20 min, sections were incubated overnight at room temperature with a mixture of the following primary antibodies (1 μg/mL each or the following dilution): anti-Car8 (1:300; RRID:AB_2571669), anti-EGFP (RRID AB_2491093), anti-EGFP (1:500; RRID:AB_10013361) and anti-vGluT1 (RRID AB_2571616) or anti-vGluT2 (1:300; RRID AB_2571620). Sections were washed and incubated for 2 h at room temperature with Alexa 488-, Cy3-, and Alexa 647-labeled species-specific secondary antibodies (Jackson ImmunoResearch; Thermo Fisher Scientific). After washing, sections were mounted on APS-coated glass slides (Matsunami, Osaka, Japan), air-dried, and coverslipped with ProLong Glass (Thermo Fisher Scientific). Confocal images were acquired using an FV1200 confocal microscope (Evident) equipped with 473, 559, and 547 nm diode lasers and UPlanXApo 60×/1.42 oil-immersion or UPlanXApo 40×/0.95 objectives. Fluorescence crosstalk was minimized by spectral slit adjustment and sequential scanning. Image analysis was performed using MetaMorph (Molecular Devices) or Fiji (ImageJ, NIH). Data were collected from L7-GFP control, ZFP64-KD, P/Q-KD, Sema3A-OE, ZFP64-KD + Sema3A-KD, and P/Q-KD + Sema3A-KD mice.

#### Double-labeling pre-embedding immunoelectron microscopy

Mice were deeply anesthetized with pentobarbital (100 μg/g body weight, intraperitoneal injection) and perfused transcardially with 4% PFA in 0.1 M PB (pH 7.2) for 10 min. The brains were then post-fixed in the same fixative for 2 h, and parasagittal sections (50-μm-thick) were cut using a VT1000S microslicer. PBS (pH 7.4) containing 0.1% Tween 20 was used for incubation and washing. Sections were blocked with 10% normal goat serum (Nichirei Bioscience Corporation) for 20 min, followed by overnight incubation at room temperature with rabbit anti-EGFP (RRID:AB_2491093) and guinea pig anti-vGluT1 (RRID:AB_2571534) antibodies (1 μg/mL each). After washing, sections were incubated with colloidal gold (1.4 nm)-conjugated anti-rabbit IgG (1:100; Nanoprobes) for 2 h at room temperature. Sections were washed in HEPES buffer (50 mM HEPES, 200 mM sucrose, pH 8.0) and incubated with silver enhancement reagent (AURION R-Gent SE-EM) for 1 h. Subsequently, sections were incubated with biotin-SP conjugated guinea pig IgG (1:200; Jackson ImmunoResearch) for 4 h, streptavidin (Nichirei Bioscience Corporation) for 30 min, and visualized with 3,3ʹ-diaminobenzidine (DAB) staining (Millipore). Sections were treated with 1% osmium tetroxide for 15 min, stained with 2% uranyl acetate for 30 min, dehydrated, embedded in Epon 812, and polymerized at 60°C for 48 h. Ultrathin sections (∼80-nm-thick) were cut using an Ultracut UCT (Leica Microsystems), stained with 2% uranyl acetate and lead citrate, and imaged at ×15,000 magnification with a JEM 1400 electron microscope (JEOL). For quantitative analysis, post-synaptic contacts labeled with EGFP were counted against PF presynaptic terminals expressing vGluT1 in electron micrographs using MetaMorph software. Data were collected from L7-GFP control, ZFP64-KD, and P/Q-KD mice.

#### Anterograde tracer labeling

Under deep anesthesia with approximately 2% isoflurane (Pfizer), a glass pipette (G-1.2; Narishige) containing 2–3 μL of 10% dextran conjugated with Alexa Fluor 594 (DTR-594; Invitrogen) in PBS was stereotaxically inserted into the inferior olive via a dorsal approach.^55^ A total of 80 nL of DTR-594 was delivered over 3 min using a syringe pump (Micro4, World Precision Instruments). After a 2-day recovery period, the mice were re-anesthetized and transcardially perfused for fixation. Data were collected from L7-GFP control, ZFP64-KD, and P/Q-KD mice.

#### Fluorescent *in situ* hybridization

Complementary DNA (cDNA) fragments of mouse ZFP64 isoform 1 (424–2454; NM_009564) and 67 kDa-glutamic acid decarboxylase (GAD67; 1035–2015; NM_008077) were subcloned into the Bluescript II plasmid vector. Digoxigenin- or fluorescein-labeled cRNA probes were produced by *in vitro* transcription as indicated in previous studies. After anesthesia with pentobarbital (100 mg/kg body weight, i.p.), fresh brains were separated from mouse skulls and instantly frozen in powdered dry ice. Fresh frozen sections were air-dried and fixed by dipping in 4% PFA in 0.1 M PB for 15 min. For immunohistochemical detection of DIG and fluorescein, sections were blocked with DIG blocking solution (TNT buffer containing 1% blocking reagent [Roche Diagnostics, Basel, Switzerland] and 4% normal sheep serum) for 30 min, and 0.5% TSA blocking reagent (PerkinElmer) in TNT buffer for 30 min. Sections were then incubated with peroxidase-conjugated anti-DIG (Roche Diagnostics; 1:1000, 1 h) or anti-fluorescein antibody (Roche Diagnostics; 1:1000, 1 h) for fluorogenic detection. After twice washing in TNT buffer for 15 min, the first detection was performed with peroxidase-conjugated anti-fluorescein antibody, followed by incubation with the FITC-TSA plus amplification kit (PerkinElmer). After the inactivation of residual peroxidase activity by dipping sections in 1% H_2_O_2_ for 30 min, the second detection was performed by incubating sections in DIG-labeled cRNA probe, followed by peroxidase-conjugated anti-DIG antibody, and the Cy3-TSA plus amplification kit (PerkinElmer). Images of fluorescent *in situ* hybridization were captured using a confocal laser-scanning microscope (FV1200; Olympus, Tokyo, Japan) quipped with 473, 559, and 647 diode laser lines, and UPlanSApo (10×/0.40) and UPlanSApo (20×/0.75) objective lenses (Olympus). TOTO3 (Thermo Fisher Scientific) was used for fluorescent nuclear counterstaining.

#### RNA sequencing

Mice at P11 with their cerebella transfected with the L7-GFP vector (control) or the L7-ZFP64-miR (1, 2)-GFP vectors (ZFP64-KD) were used. GFP-positive cerebellar tissues were collected from the GFP-expressing regions of cerebellar slices that were carefully dissociated from the non-fluorescent regions in the ACSF bubbled with 95% O_2_ and 5% CO_2_. Total RNA was extracted from the GFP-positive cerebellar tissues using RNeasy mini kit (QIAGEN) and Sepasol-RNA I Super G (Nacalai Tesque). The sequencing of messenger RNAs and the gene expression analysis were performed by GENEWIZ (Azenta). The RNA of each sample was quantified and qualified by Agilent 2100 Bioanalyzer (Agilent Technologies, Palo Alto, CA, USA), and NanoDrop (Thermo Fisher Scientific Inc.). Four samples from either control or ZFP64 KD cerebella containing more than 1 μg of total RNA (RIN value above 7) were utilized for the following library preparation via NEBNext Ultra Directional RNA Library Prep Kit for Illumina. Complementary DNAs were sequenced with an Illumina HiSeq instrument (Illumina) and the sequenced data were investigated by the fragments per kilobase of exon per million reads mapping (FRKM). Differential expression analysis was carried out using the DESeq Bioconductor package. Differentially expressed (DE) genes between control and ZFP64-KD mice were attained with a threshold of log (fold change) ± 0.58 (<0.67, >1.5 times against control) and of the adjusted *p*-value (*q*-value) smaller than 0.05. Gene ontological enrichment analysis and protein-protein interaction network analysis of the up- and down-regulated DEGs were performed using STRING version 12 (https://string-db.org). The line thickness of the network indicates the strength of data support over the medium confidence score (>0.4) about both functional and physical protein associations. *p*-values in each enrichment analysis were adjusted through Benjamini and Hochberg’s approach for *q*-value.

### Quantification and statistical analysis

All statistical values were expressed as the mean ± SEM. Because CF-EPSC step numbers are discrete, limited in range (1, 2, 3, and ≥4), and their distributions are nonparametric, the Mann-Whitney *U* test was used for two-group comparisons, and the Kruskal-Wallis test followed by the Steel-Dwass post-hoc test was applied for comparisons among three or four groups. For the two-group comparison of data that followed normal distributions (checked by the Shapiro-Wilk test), Student’s *t* test was used. These include the CF-EPSC kinetics and the frequency and amplitude of mIPSCs. One-way ANOVA with Tukey’s post-hoc test was used for evaluating the KD efficacy, where the data followed a normal distribution. To compare the PF-EPSC input-output relationship between the control and KD groups, two-way repeated measures ANOVA with Tukey’s post-hoc test was applied. For the quantitative analysis of immunofluorescence data, the Mann-Whitney *U* test was used for two-group comparisons, and the Kruskal-Wallis test followed by Steel-Dwass post-hoc test was applied for three-group comparisons, because the data for vGluT2 signal quantification included those with non-parametric distributions. For vGluT1 signal quantification, one-way ANOVA with Tukey’s post-hoc test was used because the data followed normal distributions (checked by using the Shapiro-Wilk test). For the quantitative analysis of electron microscopic data, the Kruskal-Wallis test followed by the Steel-Dwass post-hoc test was used because the data were discrete and a few in value 8. Statistical analyses were performed using EZR,^50^ which is a modified version of R (The R Foundation) and GraphPad Prism software (GraphPad software). Differences between or among groups were considered statistically significant when *p-*values were less than 0.05.
