# Supplementary material for: The transcription factor ZFP64 promotes activity-dependent synapse elimination during postnatal cerebellar development
Source: iScience. 2025 May 26;28(6):112746. doi: 10.1016/j.isci.2025.112746 (PMC12178792; doi:10.1016/j.isci.2025.112746)
Supplement: Document S1. Figures S1–S7 and Tables S1–S3 [file mmc1.pdf]

iScience, Volume *28*

Supplemental information

The transcription factor ZFP64 promotes activity-dependent synapse elimination during postnatal cerebellar development

Jianling Zhang, Takaki Watanabe, Taisuke Miyazaki, Miwako Yamasaki, Kohtarou Konno, Yuto Okuno, Kyoko Matsuyama, Takayuki Noro, Masahiko Watanabe, Naofumi Uesaka, and Masanobu Kano

Figure S1

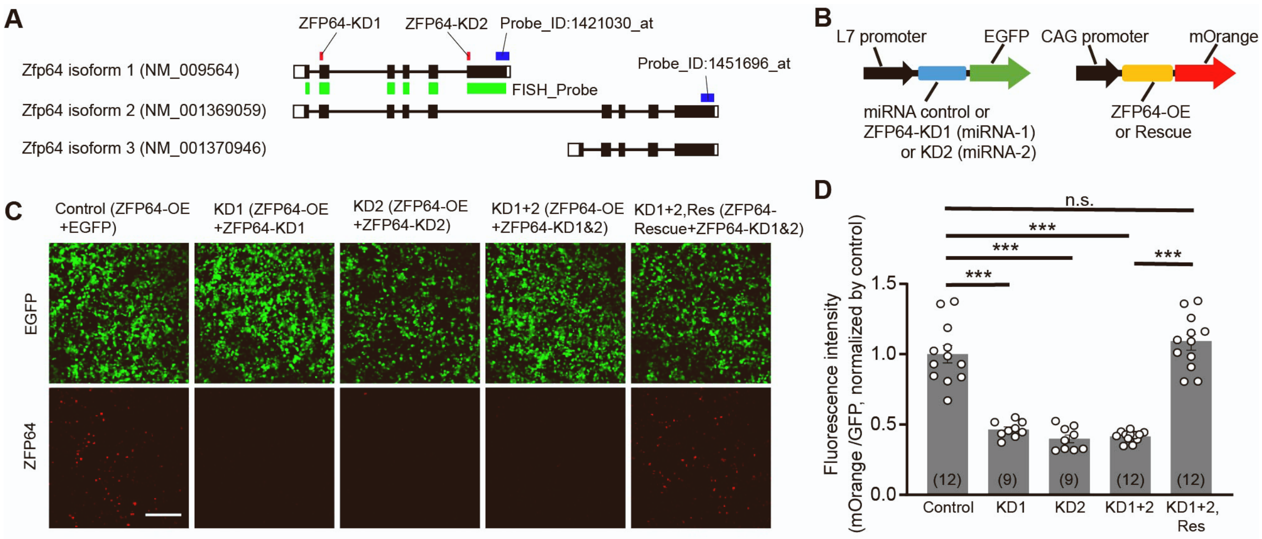

Figure S1. Transcript structures of the *Zfp64* gene and the efficacy of ZFP64 knockdown, related to Figure 1-6.

(**A**) Schema of transcript structures of *Zfp64* gene showing the isoform 1, 2, and 3 (black box, exon; white box, untranslated region). Blue bars show the probes used for microarray analysis in Table S1. Green bars show the anti-sense probe used for fluorescent *in situ* hybridization (FISH) in Figure 1A and B. Red bars indicate *Zfp64*-miRNA target 1 (KD1) and target 2 (KD2). (**B**) Schema of the vector construct for control or ZFP64-KD with EGFP (left) and for the expression of ZFP64 isoform 1 (ZFP64-OE) or miRNA-resistant ZFP64 (ZFP64-Rescue) whose cDNA was designed to be resistant to both ZFP64 miRNA-1 and miRNA-2 with mOrange (right). (**C**) Fluorescent image of HEK293T cells transfected with indicated constructs. Scale bar, 200 µm. (**D**) Summary graph showing the mOrange intensity relative to the GFP intensity in control cells (Control), cells expressing ZFP64 miRNA-1 (KD1), cells expressing ZFP64 miRNA-2 (KD2), cells expressing both miRNA-1 and 2 (KD1+2), and cells coexpressing KD1+2 with a miRNA-resistant form of ZFP64 (KD1+2, Res). The sample numbers of the observed area are shown in parentheses. One-way ANOVA, $F_{(4, 49)} = 62.3$, $p < 0.001$, followed by Tukey's test, $p < 0.001$ (Control vs KD1), $p < 0.001$ (Control vs KD2), $p < 0.001$ (Control vs KD1+2), $p = 0.599$ (Control vs KD1+2, Res), $p = 0.864$ (KD1 vs KD2), $p = 0.939$ (KD1 vs KD1+2), $p < 0.001$ (KD1 vs Res), $p = 0.999$ (KD2 vs KD1+2), $p < 0.001$ (KD2 vs Res), and $p < 0.001$ (KD1+2 vs Res) . ***$p < 0.001$, n.s. $p > 0.05$. Data are represented as mean ± SEM.

Figure S2

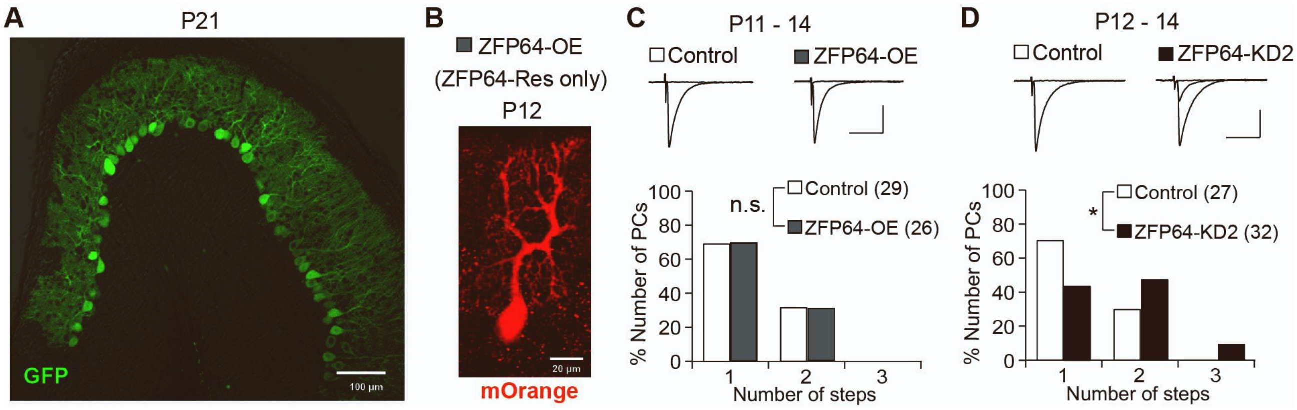

Figure S2. The effect of ZFP64 overexpression and the isoform 1 knockdown on CF synapse elimination, related to Figure 1.

(**A**) Low magnification image of the ZFP64-KD-infected cerebellum at P21. Scale bar, 100 µm. (**B**) Confocal image showing an mOrange-expressing PC at P12, infected with the ZFP64-P2A-mOrange lentivirus (ZFP64-Res only, i.e., ZFP64-overexpression (OE)). Scale bar, 20 µm. (**C, D**) CF innervation of PCs during P11-14 (**C**) and P12-14 (**D**). (Upper panels) Representative traces of CF-EPSCs recorded from GFP/mOrange-negative untransfected control PCs (white, **C, D**), mOrange-positive ZFP64-OE PCs (gray, **C**), and GFP-positive ZFP64-KD2 PCs (black, **D**). Holding potential, −10 mV. Scale bars, 1 nA and 20 ms. (Lower panels) Frequency distribution histograms for the number of CFs innervating individual PCs. The sample numbers of PCs are shown in parentheses. Mann-Whitney *U* test, (**C**) p = 0.962 and (**D**) p = 0.028. *p < 0.05, n.s. p > 0.05.

Figure S3

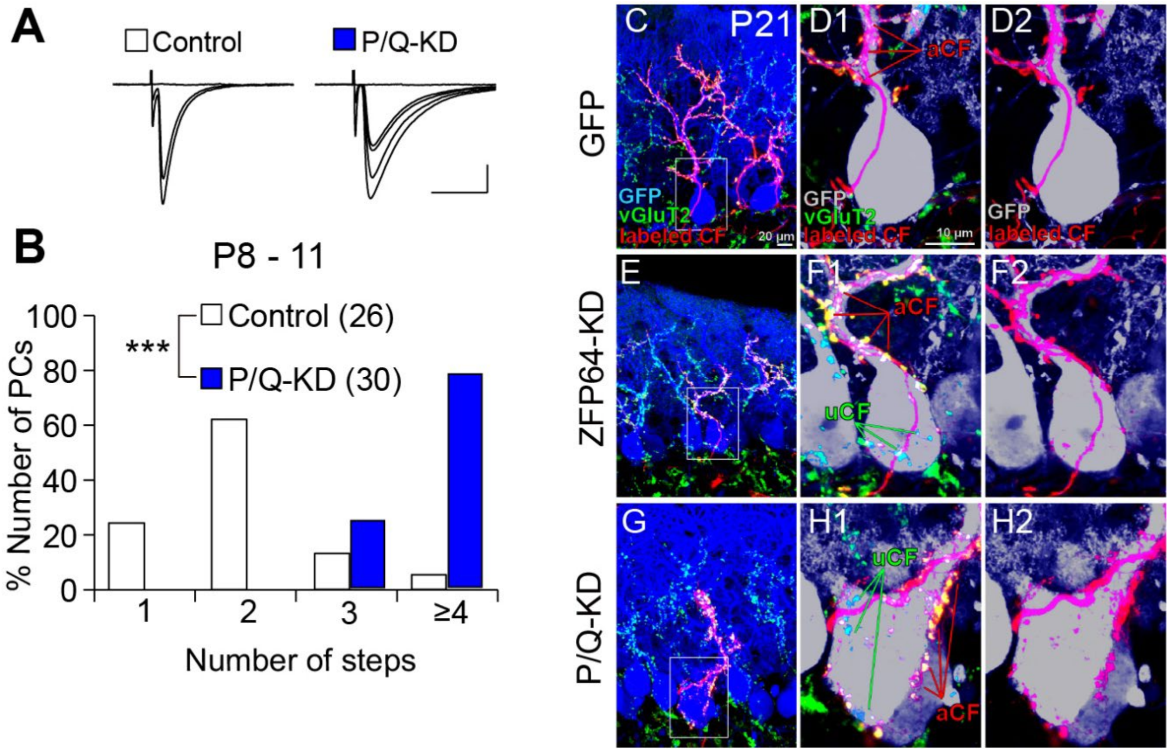

Figure S3. Impaired early phase of CF elimination in P/Q-KD PCs and morphological evidence for multiple CF innervation in ZFP64-KD PCs at P21, related to Figures 2-4 and 6.

(**A**) Representative CF-EPSCs from control (left) and P/Q-KD (right) PCs. Scale bars, 1 nA and 20 ms. (B) Frequency distribution histograms showing the number of CFs innervating each PC during P8-11 for control (white) and P/Q-KD (blue) PCs. Mann-Whitney *U* test, $***p < 0.001$. (**C-H**) Triple fluorescent labeling for GFP (blue/gray), vGluT2 (green), and anterograde tracer DTR (red) of the cerebellum from GFP control mice (**C, D**), ZFP64-KD mice (**E, F**) and P/Q-KD mice (**G, H**) at P21. “aCF” and “uCF” indicate “anterogradely labeled CF” and “anterogradely unlabled CF”, respectively. Scale bars, 20 μm (**C, E, G**), 10 μm (**D, F, H**).

Figure S4

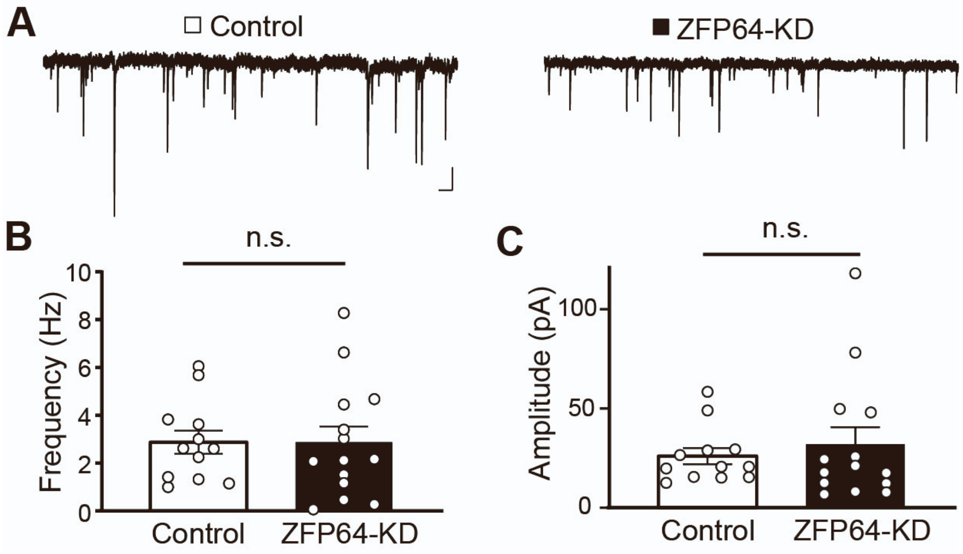

Figure S4. ZFP64-KD in PCs does not affect inhibitory synaptic responses, related to Figure 4.

(**A**) Sample traces of mIPSCs recorded from a control GFP-negative PC (left) and a GFP-positive ZFP64-KD PC (right). The holding potential was −70 mV. Scale bars, 50 pA and 200 ms. (**B, C**) Summary bar graphs representing the frequency (**B**) and amplitude (**C**) of mIPSCs for control (white, n = 12) and ZFP64-KD (black, n = 14) PCs from mice at P10 to P14. Student's *t* test, (**B**) $p = 0.998$ and (**C**) $p = 0.553$. n.s. $p > 0.05$. Data are represented as mean ± SEM.

Figure S5

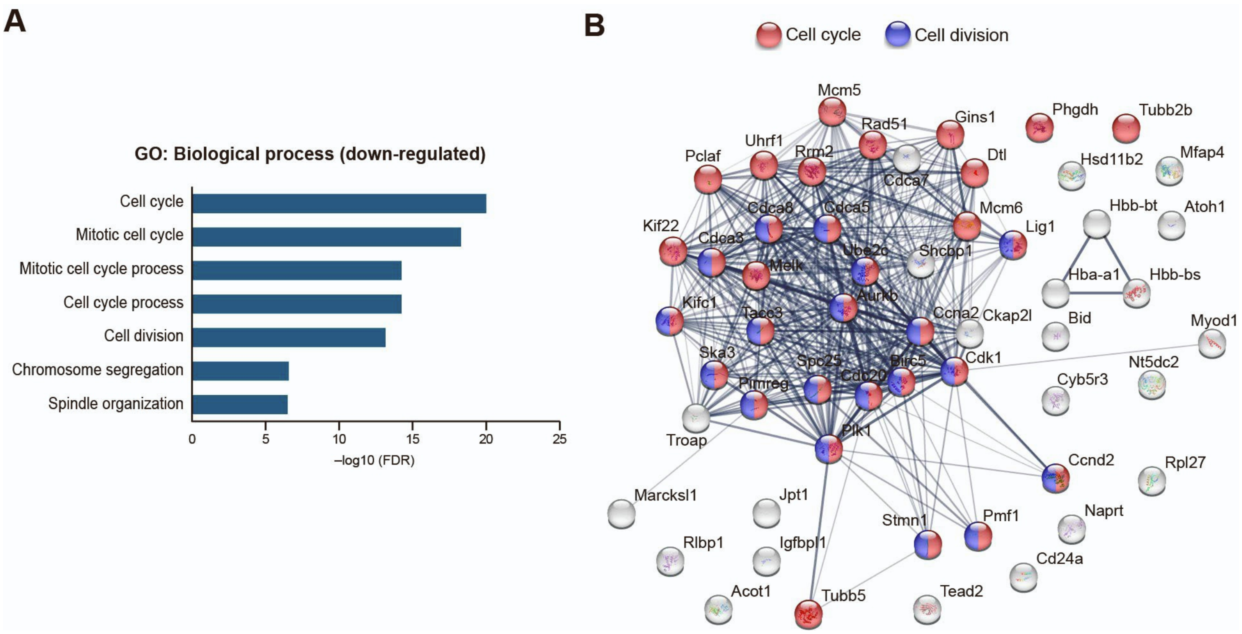

Figure S5. Down-regulated differentially expressed genes (DEGs) in the mouse cerebellum with ZFP64-KD in PCs compared to that with GFP expression in PCs, related to Figure 5.

(**A**) Gene ontology (GO) annotations related to biological processes associated with down-regulated DEGs in the cerebellum with ZFP64-KD in PCs. (**B**) Protein-protein interaction network analysis of down-regulated DEGs. Network analysis was performed using the STRING database.

Figure S6

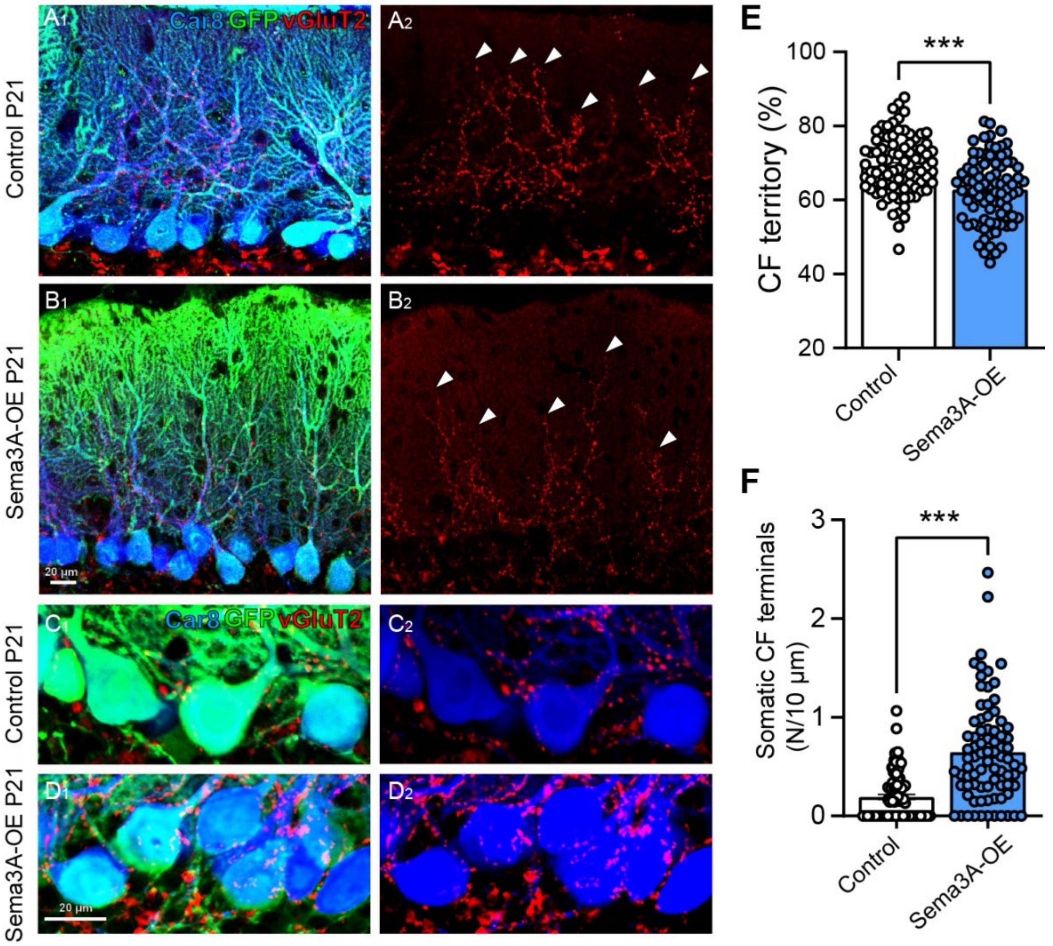

Figure S6. Sema3A-OE causes surplus CF terminals on Purkinje cell soma and impaired dendritic translocation of CFs, related to Figure 6.

(**A-D**) Triple fluorescent labeling for Car8 (blue), GFP (green), and vGluT2 (red) of the cerebellum from control mice and mice with Sema3A-OE in PCs at P21. Control mice were injected with the lentivirus carrying L7-GFP vector (**A, C**), whereas mice with Sema3A-OE in PCs were transfected with the lentivirus containing L7-Sema3A-P2A-GFP and L7-GFP vectors (**B, D**). Scale bars, 20 μm. Arrowheads show CF terminals along PC dendrites in control and Sema3A-OE mice (**A, B**). Multiple CF terminals remained around PC somata in Sema3A-OE mice (**C, D**). (**E, F**) Summary bar graphs representing the relative height of vGluT2 signals to the molecular layer thickness (E, n = 178 for Control, n = 168 for Sema3A-OE) and the number of vGluT2 terminals per 10 μm along GFP-expressing PC somata (F, n = 92 for control, n = 91 for Sema3A-OE) in GFP control and Sema3A-OE mice at P21. Mann-Whitney *U* test, (**E**, **F**) ***$p < 0.001$. Data are represented as mean ± SEM.

Figure S7

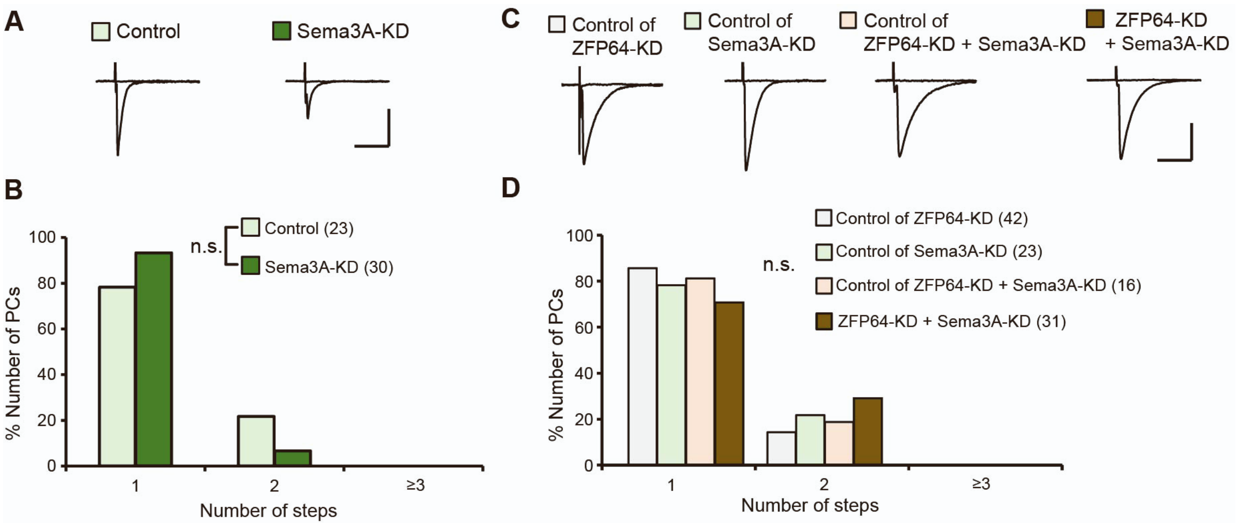

Figure S7. Sema3A-KD in PCs does not affect CF innervation patterns after weaning, related to Figure 6.

(**A**) Sample traces of CF-EPSCs recorded from a control (left), and a Sema3A-KD (right) PC. Scale bars, 1 nA and 20 ms. (**B**) Frequency distribution histogram showing the number of CFs innervating each PC during P19-30 for control and Sema3A-KD PCs. Mann-Whitney *U* test, $p = 0.112$. (**C, D**) Sample traces of CF-EPSCs (**C**) and frequency distribution histograms showing the number of CFs innervating each PC for GFP-negative untransfected PCs sampled in the same slices containing ZFP64-KD PCs (light purple), Sema3A-KD PCs (light green), and ZFP64-KD + Sema3A-KD PCs (light orange), and ZFP64-KD + Sema3A-KD PCs (brown), respectively. No significant difference was found among the four groups (Kruskal-Wallis test, $H_{(3)} = 2.4$, $p = 0.492$). The sample numbers of PCs are shown in parentheses. Scale bar, 1 nA and 20 ms. n.s. $p > 0.05$.

Table S1

Normalized expression by GAPDH	P4	P6	P7	P9	P13	P15
**Zfp64 isoform 1 (Probe_ID:1421030_at)**	0.035 (present)	0.030 (present)	0.035 (present)	0.024 (present)	0.020 (present)	0.028 (present)
**Zfp64 isoforms 2 and 3 (Probe_ID:1451696_at)**	0.0002 (absent)	0.0006 (absent)	0.0001 (absent)	0.0031 (absent)	0.0012 (absent)	0.0024 (absent)

Table S1. Relative expression of ZFP64 transcripts in PCs during postnatal development
Expression levels of ZFP64 mRNA by using the probes for isoform 1 and isoforms 2 and 3 are normalized to glyceraldehyde-3-phosphate dehydrogenase (GAPDH). The presence or absence shown in parentheses was determined by a detection algorithm of probe pair (perfect match/mismatch) intensities. Data were derived from the DNA microarray analysis using purified Purkinje cells from L7-GFP transgenic mice.

Table S2

		CF group	Amplitude (nA)	10-90% rise time (ms)	Decay time constant (ms)
**P8-11**	Control	CF-mono	2.57 ± 0.45 (14)	0.56 ± 0.03 (10)	4.77 ± 0.68 (10)
		CF-multi-S	1.47 ± 0.23 (19)	0.59 ± 0.04 (8)	5.22 ± 0.70 (8)
		CF-multi-W	0.55 ± 0.14 (25)	0.48 ± 0.02 (12)	4.01 ± 0.28 (13)
	ZFP64-KD	CF-mono	3.61 ± 0.51 (7)	0.59 ± 0.02 (6)	5.01 ± 0.61 (6)
		CF-multi-S	1.02 ± 0.09 (5)	0.53 ± 0.03 (6)	4.59 ± 0.48 (8)
		CF-multi-W	0.41 ± 0.04 (33)	0.49 ± 0.03 (11)	3.84 ± 0.41 (12)
**P13-15**	Control	CF-mono	2.45 ± 0.20 (10)	0.58 ± 0.04 (10)	6.28 ± 0.66 (10)
		CF-multi-S	1.75 ± 0.39 (6)	0.51 ± 0.07 (3)	6.74 ± 1.46 (3)
		CF-multi-W	0.53 ± 0.12 (14)	0.51 ± 0.04 (5)	3.67 ± 0.48 (5)
	ZFP64-KD	CF-mono	2.08 ± 0.38 (8)	0.69 ± 0.06 (4)	6.03 ± 0.55 (6)
		CF-multi-S	1.70 ± 0.35 (10)	0.67 ± 0.14 (3)	5.91 ± 0.43 (3)
		CF-multi-W	0.53 ± 0.12 (14)	0.51 ± 0.04 (5)	3.67 ± 0.48 (5)
**P19-30**	Control	CF-mono	2.50 ± 0.21 (25)	0.53 ± 0.04 (12)	6.53 ± 0.40 (12)
		CF-multi-S	1.61 ± 0.13 (5)	0.53 ± 0.08 (3)	7.83 ± 1.50 (3)
		CF-multi-W	0.37 ± 0.11 (5)	0.47 ± 0.07 (4)	4.81 ± 1.37 (4)
	ZFP64-KD	CF-mono	2.53 ± 0.25 (16)	0.48 ± 0.05 (5)	7.14 ± 0.60 (5)
		CF-multi-S	2.09 ± 0.21 (19)	0.55 ± 0.03 (5)	8.24 ± 0.67 (6)
		CF-multi-W	0.60 ± 0.11 (21)	0.47 ± 0.04 (12)	4.60 ± 0.51 (12)

Table S2. Kinetics of the three categories of CF-EPSCs recorded from control and ZFP64 KD PCs from P8 to P30.

CF-EPSCs are divided into three groups, "CF-mono" represents CF-EPSCs from mono-innervated PCs, "CF-multi-S" and "CF-multi-W" represent the largest CF-EPSCs and the other smaller CF-EPSCs, respectively, in individual multiply innervated PCs. Sample numbers of PCs are displayed in parentheses. Data are represented as mean ± SEM. The amplitudes were recorded at a holding potential of −10 mV. Statistical *p*-values from Student's *t* test comparing Control and ZFP64 PCs are shown as follows:

P8-11: Amplitude; p = 0.171 (CF-mono), p = 0.094 (CF-multi-S), p = 0.675 (CF-multi-W), Rise time; p = 0.538 (CF-mono), p = 0.290 (CF-multi-S), p = 0.777 (CF-multi-W), Decay time constant; p = 0.815 (CF-mono), p = 0.470 (CF-multi-S), p = 0.731 (CF-multi-W).

P13-15: Amplitude; p = 0.380 (CF-mono), p = 0.931 (CF-multi-S), p = 0.590 (CF-multi-W), Rise time; p = 0.126 (CF-mono), p = 0.352 (CF-multi-S), p = 0.778 (CF-multi-W), Decay time constant; p = 0.798 (CF-mono), p = 0.615 (CF-multi-S), p = 0.367 (CF-multi-W).

P19-30: Amplitude; $p = 0.914$ (CF-mono), $p = 0.264$ (CF-multi-S), $p = 0.330$ (CF-multi-W), Rise time; $p = 0.720$ (CF-mono), $p = 0.822$ (CF-multi-S), $p = 0.974$ (CF-multi-W), Decay time constant; $p = 0.416$ (CF-mono), $p = 0.772$ (CF-multi-S), $p = 0.863$ (CF-multi-W).

Table S3

	CF group	Total amplitude (nA)	Disparity ratio	Disparity index	PPR (interval 50 ms)
**P8-11**	Control	2.45 ± 0.29 (31)	0.43 ± 0.07 (19)	0.66 ± 0.10 (19)	0.65 ± 0.02 (28)
	ZFP64-KD	2.37 ± 0.22 (25)	0.49 ± 0.05 (15)	0.54 ± 0.07 (15)	0.62 ± 0.03 (19)
**P13-15**	Control	2.43 ± 0.25 (16)	0.41 ± 0.12 (6)	0.66 ± 0.17 (6)	0.70 ± 0.03 (15)
	ZFP64-KD	2.44 ± 0.27 (19)	0.43 ± 0.08 (10)	0.65 ± 0.13 (10)	0.70 ± 0.04 (15)
**P19-30**	Control	2.41 ± 0.18 (30)	0.22 ± 0.05 (5)	0.92 ± 0.10 (5)	0.73 ± 0.01 (29)
	ZFP64-KD	2.65 ± 0.17 (35)	0.33 ± 0.06 (19)	0.78 ± 0.09 (19)	0.73 ± 0.01 (33)

Table S3. Total amplitude, disparity ratio/index, and paired-pulse ratio of CF-EPSCs from control and ZFP64 KD PCs from P8 to P30

The total amplitude, disparity parameters (disparity ratio, disparity index), and paired-pulse ratio (PPR) for CF-mono, CF-multi-S, and CF-multi-W of control and ZFP64-KD PCs from P8 to P30. Sample numbers of PCs are displayed in parentheses. Data are represented as mean ± SEM. The total amplitudes were measured at a holding potential of −10 mV. Statistical *p*-values from Student's *t* test comparing Control and ZFP64 PCs are shown as follows:

P8-11: Total amplitude; $p = 0.831$, Disparity ratio; $p = 0.572$, Disparity index; $p = 0.815$, PPR; $p = 0.337$.

P13-15: Total amplitude; $p = 0.993$, Disparity ratio; $p = 0.884$, Disparity index; $p = 0.939$, PPR; $p = 0.980$.

P19-30: Total amplitude; $p = 0.343$, Disparity ratio; $p = 0.351$, Disparity index; $p = 0.447$, PPR; $p = 0.989$.
